# Sustainable one-part geopolymeric hybrid composite derived from glauconite, talc, and olive seed waste–based activated carbon for Congo red adsorption

**DOI:** 10.1038/s41598-026-50411-7

**Published:** 2026-05-22

**Authors:** Abdelrahman G. Gadallah, Ahmed S. Elshimy, A. A. Hegazy, Mohammod H. Rahman

**Affiliations:** 1 Chemical Engineering Department, College of Engineering, Imam Mohammad Ibn Saud Islamic University (IMSIU), Riyadh, 11432 Saudi Arabia; 2 Faculty of Earth Science, Beni–Suef University, Beni, Suef, 62511 Egypt; 3 Department of Refractories, Ceramics, and Building Materials, National Research Centre, P.O. 12622, 33 El Bohooth st. (former El Tahrir st.), Dokki, Cairo, Giza Egypt

**Keywords:** One-part geopolymer composite, Olive seed waste, Congo red, Experimental analyses, Statistical physics modeling, ANOVA studies, Chemistry, Engineering, Environmental sciences, Materials science

## Abstract

This study details the synthesis and application of a novel one-part geopolymeric hybrid composite (OP-GPHC) derived from glauconite, talc, and olive seed waste–based activated carbon for the efficient sequestration of Congo red (CR) dye from contaminated water. The hybrid binder was synthesized by impregnating activated carbon-based biogenic waste into a thermally treated glauconite/talc matrix, followed by alkali-activation with NaOH. Comprehensive characterization via XRD, FTIR, BET, TG/DTG, FESEM/EDX, and elemental mapping confirmed the material’s exceptional adsorptive properties. A Box–Behnken design (BBD) optimization established the optimal operational conditions: pH 2.0, adsorbent dosage 0.07 g/ 25 ml, contact time 77.5 min, initial CR concentration 150 mg/L, and temperature 328 K, achieving a removal efficiency of 99.2%. Equilibrium data were best described by the Langmuir isotherm, yielding a maximum adsorption capacity of 367 mg/g at 328 K, while kinetic data followed the pseudo-first-order (PFO) model. Advanced statistical physics modeling revealed a multimolecular adsorption mechanism with a vertical orientation of CR molecules at the active sites, independent of temperature, and binding energies in the range of 19.25–21.46 kJ/mol, consistent with physisorption dominated by hydrogen bonding, π–π interactions, and electrostatic forces. Thermodynamic parameters confirmed the endothermic and spontaneous nature of the process. The OP-GPHC adsorbent exhibited excellent reusability (87.8% after five cycles) and a low production cost of $0.032/g. Based on batch-derived data, treatment of 100 L of CR-contaminated water (50 mg/L) is projected to cost approximately $1.68, highlighting its strong potential for industrial-scale tertiary treatment applications.

**Supplementary Information:**

The online version contains supplementary material available at 10.1038/s41598-026-50411-7.

## Introduction

In recent centuries, water pollution driven by population growth, industry, and intensive animal agriculture has become a major threat to ecosystems and human health, introducing harmful micropollutants into water^1–3^. Untreated effluents contaminate drinking water, food production, and agriculture^4,5^. Among these pollutants, non-biodegradable synthetic dyes like CR, which contains two azo groups (–N = N–), are particularly hazardous due to their bioaccumulation, toxicity, and carcinogenicity^6,7^. CR enters water mainly from textile and dyeing industries and is easily detected due to visible light absorption^2,6,8^.

Various physical, chemical, and biological methods exist for dye remediation^9^. Adsorption is favored for its effectiveness, simplicity, low cost, and minimal hazardous sludge^5,7,10^. While several adsorbents have been used for CR removal^3,11,12^, there is ongoing need for novel, sustainable adsorbents from low-cost, circular economy methods. Alkali-activated materials (geopolymers) offer a promising solution^5,8^. Geopolymers are inorganic polymers, sustainable alternatives to Portland cement^8^, with high surface area, stability, and cost efficiency^13–15^. They are typically synthesized from amorphous aluminosilicate precursors and alkaline activators^8,16–18^. One-part geopolymers (mixing water with solid activators) are safer and easier to scale than conventional two-part systems^16,17^.Calcined clays like raw glauconite (RG)—rich in K, Al, Si, Fe—and raw talc (RT)—rich in Mg with layered structure—are effective adsorbents for dyes^2,8,17–19^. Olive seed waste (OSW), a carbon-rich byproduct of olive oil production, can be converted into activated carbon, offering a low-cost, high-performance adsorbent^20,21^.

Recent geopolymer-based adsorbents have targeted various contaminants: uranium (0.236 mol kg⁻¹, Freundlich, PSO)^22^, methylene blue (76.34 mg/g, Freundlich, PSO; and 283.42 mg/g, Langmuir, PFO)^10,23^, Cr(VI) (107.56 mg/g, Langmuir, PSO)^24^. and ibuprofen (52.33 mg/g Langmuir)^25^. In the present work, a vitrification step was employed prior to alkali activation to transform the crystalline glauconite and talc precursors into amorphous, highly reactive phases. This thermal treatment at 1000 °C disrupts the original mineral structures, promoting the formation of a homogeneous glassy matrix that significantly enhances the subsequent geopolymerization reaction. Vitrification facilitates the dissolution of alumina and silica species in the solid activator medium, leading to faster setting and improved mechanical integrity of the final binder. Similar strategies have been reported in the literature for producing one-part geopolymers from various aluminosilicate sources, where pre-calcination or vitrification is used to increase reactivity and simplify the activation process^26,27^.

Crucially, the development of this material aligns with several United Nations Sustainable Development Goals (SDGs). The use of locally sourced natural clays (glauconite, talc) and an agricultural byproduct (olive seed waste) directly contribute to SDG 12 (Responsible Consumption and Production) by valorizing waste streams and promoting circular economy principles. The synthesis protocol, which avoids toxic organic solvents and relies on a one-part geopolymerization approach, minimizes environmental impact, supporting SDG 13 (Climate Action) through reduced energy consumption and carbon footprint compared to conventional adsorbent synthesis. Furthermore, the material’s exceptional efficacy in removing CR—a carcinogenic azo dye—from contaminated water provides a tangible contribution to SDG 6 (Clean Water and Sanitation), specifically target 6.3, which aims to improve water quality by reducing pollution and hazardous chemical releases. The high reusability and low production cost also support SDG 9 (Industry, Innovation, and Infrastructure) by offering a scalable, cost-effective technology for industrial wastewater treatment, while the prevention of toxic dye discharge into natural water bodies aligns with SDG 14 (Life Below Water) by protecting aquatic ecosystems from persistent organic pollutants. By explicitly connecting material design to global sustainability targets, this work provides a holistic framework for developing next-generation adsorbents that are both technologically effective and environmentally responsible^28–30^.

A significant knowledge gap persists regarding the use of carbon-enhanced, one-part geopolymer composites in wastewater treatment applications. Therefore, this study presents a novel OP-GPHC as an innovative class of green adsorbents, directly addressing the urgent need for advanced water treatment materials. The key innovation lies in the first successful integration of its components, yielding a material with a remarkable CR uptake of 367 mg/g. A multi-technique characterization (XRD, FTIR, TG/DTG, BET, FESEM, EDX) was employed to reveal the structural and surface properties responsible for this performance. The primary objectives were to optimize adsorption parameters using a Box-Behnken design (BBD), evaluate adsorption dynamics through kinetic and isotherm modeling, and elucidate the molecular-level mechanisms via monolayer physics-based modeling. Additionally, thermodynamic analysis (Gibbs free energy, internal energy, entropy) was conducted to confirm the spontaneous and endothermic nature of the process. Ultimately, this work achieves the key objective of converting agricultural solid wastes and natural raw materials into a high-performance hybrid composite with industrial potential for anionic CR dye remediation.

## Materials and methods

### Materials

OSW, obtained from local vendors, was purified by soaking in distilled water at 100 °C with constant stirring (200 rpm, 1 h). For complete organic residue removal, the material underwent acid treatment using 100 mL of 1 M HCl at 50 °C under continuous agitation at 200 rpm for 30 min. Subsequently, the sample underwent triple washing with distilled water to remove residual HCl. Purified OSW was thermally treated at 800 °C for 1 h under controlled atmosphere^31^. The resulted powder was then activated with 20 mL H₂O₂ at 50 °C for 60 min under stirring, washed thrice with distilled water, producing porous OSW-derived activated carbon (OSW-AC) with high surface area^32^. The RT sample used in this study, with particle sizes of about 80 μm, was obtained from Abu Gurdi Valley (Red Sea, Egypt). Additionally, the RG sample, also with particle sizes under 100 μm, was sourced from the Central Metallurgical Research and Development Institute (CMRDI) in Egypt. The chemical compositions of RT and RG, as determined by XRF analysis, are detailed in Table 1. All chemical reagents were of analytical grade. Hydrochloric acid (HCl), sodium hydroxide (NaOH), and CR dye (C₃₂H₂₂N₆Na₂O₆S₂, molecular weight 696.665 g/mol) were procured from Adent Chembio Pvt. Ltd. in India. ZA Chem (California, USA) supplied the 50% (w/w)aqueous hydrogen peroxide solution. All chemical reagents were used without further purification. The pH of experimental solutions was calibrated as required with standardized NH₄OH or HCl.

**Table 1:** RT and RG compositions as measured by XRF.

Notations	CaO	MgO	SiO_2_	Al_2_O_3_	Fe_2_O_3_	Na_2_O	SO_3_	K_2_O	TiO_2_	*P*_2_O_5_	Cl-	LOI	Total
RT	1.07	31.79	55.68	1.38	0.83	0.22	0.08	0.01	0.02	0.02	0.05	8.82	99.97
RG	0.1	2.26 31.79	50.3	3.49	27.51	0.35	0.94	5.38	0.24	0.31	0.24	8.86	99.98

### Preparation of the OP-GPHC binder

**Fig. 1:**
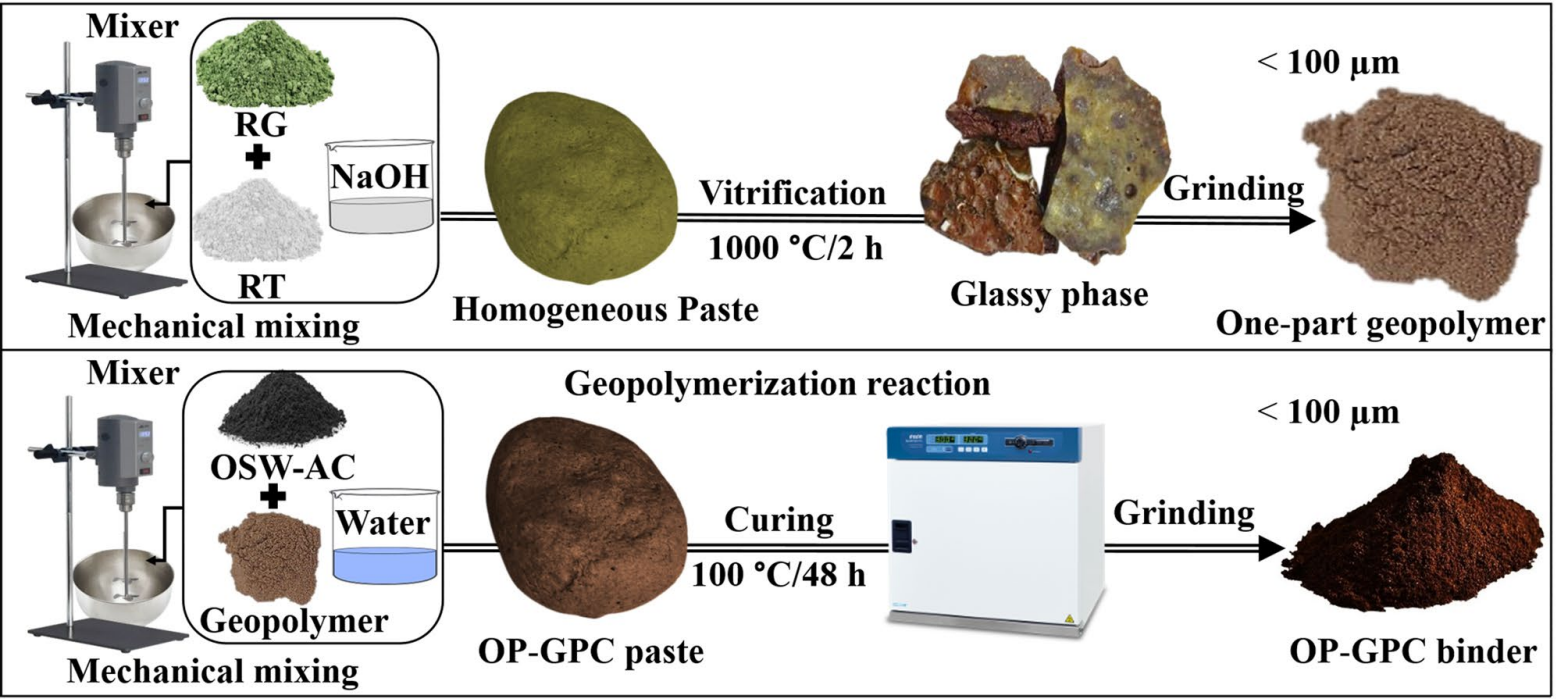
Flow diagram explaining the creation of the OP-GPHC adsorbent.

The OP-GPHC adsorbent was synthesized through a sequential preparation process. First, 20 g of RG was homogenized with 10 g of RT, then blended with 10 mL of NaOH solution containing 15% Na₂O (relative to the total powder weight of 30 g) at a mixing speed of 100 rpm via a mechanical mixer for 3 min until complete homogenization was achieved. The resulting mixture was then moved to a refractory crucible and heat-treated at 1000 °C under controlled conditions: a heating rate of 20 °C/min for 2 h under ambient air atmosphere. To maximize geopolymerization efficiency, the precursors (RG and RT) were first converted into amorphous, reactive phases via vitrification. The key vitrification parameters, specifically Na₂O concentration and processing temperature, were optimized based on previous experimental findings^17,33^. After furnace cooling, the fired product was dry-ground and sieved to obtain the final vitrified one-part geopolymer. Finally, the OP-GPHC adsorbent was synthesized by uniformly blending 10 g of vitrified one-part geopolymer powder with 2 g of OSW-AC, followed by wet mixing with tap water at a 0.5 water-to-powder ratio. Homogenization was achieved using a mechanical mixer operating at 100 rpm for 3 min to facilitate the geopolymerization process. The homogeneous paste was exposed to thermal curing in an oven at 100 °C for 48 h to accelerate the geopolymerization reaction kinetics. After curing, the hardened binder (i.e., OP-GPHC) was cooled to ambient temperature, mechanically ground, and sieved to obtain a powdered adsorbent with a particle size below 100 μm. This powder was then used for batch CR adsorption experiments Fig. 1.

### Characterization of the developed OP-GPHC binder

To elucidate the properties of the synthesized OP-GPHC adsorbent, an array of state-of-the-art analytical methods was employed. Specifically, the chemical makeup of the raw precursors (RT and RG) was analyzed via X-ray fluorescence (XRF) spectrometry using a PANalytical Axios sequential wavelength-dispersive XRF instrument (2005 model). A PANalytical Empyrean X-ray diffractometer was employed for mineralogical analysis of the RT, RG, OSW-AC, and OP-GPHC specimens. The instrument configuration utilized Cu-Kα radiation (λ = 0.154 nm) with an operating voltage and current of 40 kV and 30 mA, respectively. Diffraction patterns were recorded across a 2θ angular range from 5° to 80°, employing a scanning speed of 5° per minute. A Bruker Optics VERTEX 70 spectrometer was utilized to perform Fourier Transform Infrared Spectroscopy (FTIR) analysis, aiming to characterize the functional moieties on the sample surfaces. The investigation encompassed the raw precursors (RT, RG), the processed carbons (OSW-AC, OP-GPHC), and the post-adsorption material (CR-loaded OP-GPHC). Moreover, spectral data were collected over the 400–4000 cm⁻¹ mid-IR range to elucidate the molecular vibrations indicative of specific chemical bonds. The thermal degradation behavior and compositional profile of OP-GPHC composite powder were evaluated by thermogravimetric and derivative thermogravimetric analysis (TG/DTG). Utilizing a Stream Instrumentation (France) analyzer, samples were subjected to a thermal ramp from 30 to 830 °C at 10 °C/min under a nitrogen purge (50 mL/min). This methodology provided quantitative data on mass loss, enabling the characterization of hydrated phases and the assessment of the adsorbent’s decomposition kinetics and thermal stability in an inert atmosphere. The morphological and microstructural features of the OP-GPHC adsorbent were examined via field emission scanning electron microscopy (FESEM) using a Thermo Fisher Scientific Quanta FEG 250 instrument. Complementary energy-dispersive X-ray spectroscopy (EDX) and elemental mapping provided concomitant analysis of its spatial elemental distribution and chemical constitution. Furthermore, the textural characteristics, including the specific surface area, were determined by applying the Brunauer-Emmett-Teller (BET) method with a Micromeritics TriStar II 3020 analyzer.

### CR adsorption experiments

A series of batch adsorption experiments quantified the efficacy of the OP-GPHC biocomposite for sequestering CR from aqueous media. To evaluate their impact on the removal mechanism, key operational variables were modulated, including biosorbent mass (0.01–0.07 g/25 ml), interaction period (5–150 min), initial CR concentration (25–350 mg/L), and the critical parameter of solution acidity, investigated across a wide pH spectrum (2–9). These investigations were conducted under controlled conditions within 50 mL centrifuge tubes to isolate and accurately determine the contribution of each variable to the overall sequestration efficiency. Furthermore, the material’s adsorption capacity was characterized by deriving its isotherm profiles through exposure to CR solutions with initial concentrations spanning 25 to 350 mg/L. A standardized protocol was employed for these tests and the accompanying kinetic analysis, maintaining fixed parameters of pH 2.0, 0.02 g of biosorbent, and a 25 mL solution volume over a one-hour duration. To investigate the influence of thermal energy on the adsorption process, the isotherm studies were repeated at temperatures of 25, 40, and 55 °C. Kinetic data were generated at 25 °C with an initial CR concentration of 150 mg/L; aliquots were extracted at specified time points across the 5 to 150-min range. A constant agitation speed of 150 rpm ensured uniform mixing in all experiments. Following the adsorption phase, the mixtures were centrifuged, and the resulting supernatant was analyzed via spectrophotometry to determine the remaining dye concentration. The corresponding adsorption capacities at equilibrium ($\:{q}_{e}$) and at time $\:t$ ($\:{q}_{t}$) were subsequently calculated using Eq. (1) and the same expression with $\:{C}_{t}$ in place of $\:{C}_{e}$.

1 $$\:{q}_{e}=({C}_{O}-{C}_{e})\frac{V}{m}$$

In this context, $\:{C}_{0}$ and $\:{C}_{e}$ are the initial and equilibrium liquid-phase concentrations (mg/L), $\:V$ is the solution volume (L), and $\:m$ is the mass of the OP-GPHC adsorbent (g).

### Modeling of CR removal on OP-GPHC

A comprehensive mechanistic interpretation of adsorption-based water purification necessitates moving beyond simple efficiency measures to critically examine the physical and chemical parameters obtained from equilibrium modeling. The Langmuir and Freundlich isotherms serve as fundamental instruments in this pursuit, valued for their ability to correlate with diverse experimental results and yield preliminary data on adsorption potential and surface characteristics^34–38^. These models, however, predominantly furnish a macroscopic and phenomenological viewpoint. Elucidating the core adsorbent-adsorbate interactions that occur at the molecular scale requires the implementation of advanced statistical physics models to uncover details such as the energy distribution of active sites and the stoichiometry of the adsorption reaction^39^. By offering a more refined, energy-centric characterization of the interfacial region, these sophisticated theoretical frameworks address the shortcomings of classical methods. The rigorous validation of a combined approach, which integrates both classical and statistical physics-based models with experimental evidence, therefore permits a truly multi-scale analysis of the adsorption mechanism. This methodology effectively connects observed uptake behavior with events at the nanoscale. The complete computational protocols for these isotherm and kinetic modeling are documented in the supplementary materials.

### Thermodynamic analysis for CR removal

The thermodynamic parameters of entropy, Gibbs free energy, and internal energy serve as fundamental indicators for macroscopic characterization of the CR/OP-GPHC interfacial system under varying thermal conditions. These quantitative descriptors provide critical evidence regarding both the thermodynamic favorability and molecular compatibility of the adsorption phenomenon^38^. A comprehensive account of the thermodynamic calculations is provided in the supplementary materials.

### Statistical optimization methodology

This study identified solution pH, adsorbent dosage, and contact duration as the primary factors influencing the adsorption of CR dye. The parameter optimization and evaluation were conducted using Response Surface Methodology (RSM) implemented via a BBD, using Design-Expert software (Version 12.0, Stat-Ease Inc.). By employing mathematical and computational techniques, RSM significantly decreases the number of experimental trials required to investigate all relevant factors and their interactions. RSM comprises three primary stages: experimental design, response surface modeling, and visualization of results^40^. RSM utilizes regression analysis to quantitatively define the mathematical correlation between manipulated independent variables (Xi) and their corresponding system response (Y)^41^. The interaction is typically modeled using a quadratic function, providing a robust mathematical foundation for interpreting the experimental results. The proposed model accounts for all variables through first- and second-order terms, capturing both linear and nonlinear effects of the investigated parameters. The mathematical formulation (Eq. 2) is expressed as^41^:

2 $$\:\mathrm{Y}=\:{{\upbeta\:}}_{0}+{\sum\:}_{\mathrm{j}=1}^{\mathrm{k}}{{\upbeta\:}}_{\mathrm{j}}{\mathrm{x}}_{\mathrm{j}}+{\sum\:}_{\mathrm{j}=1}^{\mathrm{k}}{{\upbeta\:}}_{\mathrm{j}\mathrm{j}}{\mathrm{x}}_{\mathrm{j}}+\sum\:_{\mathrm{i}}{\sum\:}_{<\mathrm{j}=2}^{\mathrm{k}}{{\upbeta\:}}_{\mathrm{i}\mathrm{j}}{\mathrm{x}}_{\mathrm{i}}{\mathrm{x}}_{\mathrm{j}}+{\mathrm{e}}_{\mathrm{i}}$$

where, Y denotes the response variable that indicates the efficacy of CR uptake. The model parameters comprise k independent variables (x_i_, x_j_; i, j = 1-k), where β₀ denotes the intercept, and β_j_, β_jj_, and β_ij_ correspond to the linear, quadratic, and interaction coefficient terms, respectively. The model formulation considers k independent factors, with ε denoting the residual error term. As detailed in Table 2, the three-variable BBD examined pH (A), adsorbent dosage (B, g/L), and contact duration (C, min) across three coded levels (−1, 0, + 1), representing minimum, central, and maximum values respectively.

**Table 2:** Parameters and their levels in the BBD optimization for the CR adsorption.

Factor	Name	Units	Ranges	Minimum	Maximum	Mean
A	Aqueous pH		2–9	2	10	5.5
B	Adsorbent dosage	g	0.01–0.07	0.01	0.04	0.07
C	Interaction time	min	5–150	5	150	77.5

### Reusability of OP-GPHC adsorbent

The regeneration efficiency of the OP-GPHC adsorbent was evaluated at 25 °C by desorbing CR-loaded samples in 100 mL of HCl under continuous agitation at 200 rpm for 60 min via a rotary shaker, thereby assessing both contaminant release and adsorbent reusability. The OP-GPHC binder underwent five consecutive adsorption-desorption cycles, with intermediate regeneration involving deionized water rinsing and thermal treatment (80 °C, 24 h), while removal efficiency was systematically monitored after each cycle to assess reusability. The behavior of the OP-GPHC was assessed using the following equation (Eq. 3):

3 $$\:\:CR\:removal\:efficiency\:\left(\%\right)=\frac{{C}_{0}-{C}_{e}}{{C}_{0}}\times\:100$$

## Results and discussion

### Characterization of raw and synthesized materials

**Fig. 2:**
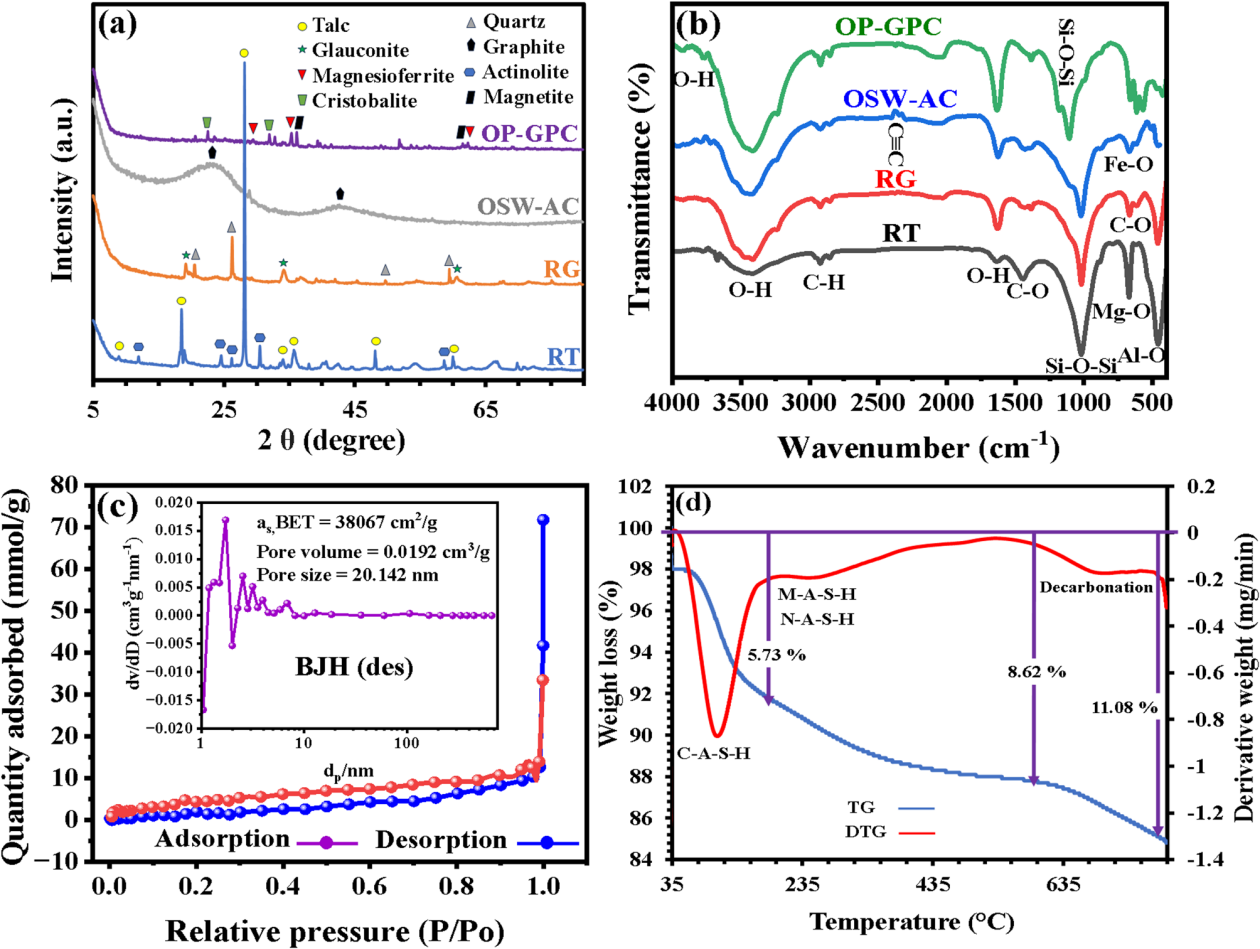
(**a**) XRD patterns and (**b**) FTIR spectra of RT, RG, OSW-AC, and OP-GPHC, where (**c**) BET studies and (**d**) TG/DTG analysis of OP-GPHC composite.

Figure 2a presents a comparative analysis of mineralogical and phase compositions derived from XRD technique, examining geomaterials (RT, RG), OSW-AC, and the synthesized OP-GPHC binder. The diffraction peaks of the RT sample revealed prominent peaks at 2θ values of 9.54º, 18.76º, 28.27º, 35.98º, 48.25º, and 60.23º, which signifies the presence of talc as a main mineral^17,42^. Additionally, actinolite was identified as a coexisting mineral, exhibiting identified peaks at 12.14º, 24.87º, 26.31º, and 58.73º^5,37^. The analysis of the RG sample demonstrated clear diffraction patterns observed at 2θ of 19.35º, 34.32º, and 60.98º, which are indicative of the glauconite mineral^2^. Furthermore, additional peaks detected at approximately 20.56º, 26.53º, 37.21º, 39.34º, 59.81º, and 67.87º were attributed to quartz mineral impurities associated with glauconite^8^. The treated OSW-AC sample exhibited two broad diffraction peaks at 2θ = 23.57º and 2θ = 42.95º, which are characteristic of the graphite phase^43^. Post-vitrification process, the XRD analysis unequivocally confirmed the total disappearance of crystalline-phase diffraction peaks in the RT and RG samples, providing definitive evidence of complete structural amorphization (vitrification) as shown in the diffraction pattern of OP-GPHC. The elevated MgO content (32.79 wt%) in the RT, combined with the high Fe₂O₃ concentration (27.51 wt%) in RG, synergistically facilitated the crystallization of magnesioferrite (MgFe₂O₄) at 1000 °C. This phase formation was unambiguously confirmed by distinct XRD reflections at 2θ angles of 29.92°, 35.41°, and 62.93°^44^. A secondary phase, magnetite (Fe₃O₄), formed through iron oxidation sourced from RG sample during thermal treatment, as confirmed by XRD peaks at 2θ = 36.26° and 61.93°^45^. Also, the crystalline silicate phase cristobalite (SiO₂) was identified through characteristic XRD reflections at 22.53° and 32.11°^46^. Following the geopolymerization process, the surplus silica and alumina found in both RT and RG (as detailed in Table 1) played a crucial role in the formation of amorphous aluminum silicate phases during vitrification, thereby significantly accelerating the kinetics of the hydration process. The geopolymerization reactions yield aluminum silicate hydrate compounds, notably sodium aluminum silicate hydrate (N-A-S-H), calcium aluminum silicate hydrate (C-A-S-H), and magnesium aluminum silicate hydrate (M-A-S-H), due to the presence of Na₂O, CaO, and MgO in the raw materials of the fabricated binder (Table 1). These compounds exhibit a fundamentally amorphous structural configuration^47^. The N-A-S-H gel phase in geopolymer systems exhibits predominantly amorphous characteristics when analyzed by X-ray diffraction^48^. Meanwhile, C-A-S-H demonstrates a structurally disordered, glass-like configuration at the atomic scale, though its amorphous nature is derived from the statistical averaging of local atomic arrangements^47^. Similarly, M-A-S-H is well-established as an amorphous phase, particularly during its early formation, where it frequently coexists with non-crystalline silica^49^. Although XRD analysis failed to conclusively identify the hydration products, their existence was irrefutably established through an integrated analytical methodology combining FTIR spectroscopy, FESEM, and EDX coupled with elemental mapping analyses.

The FTIR spectra of RT, RG, OSW-AC, and OP-GPHC is presented in Fig. 2b. The observed broad absorption peaks at 1638.23 cm⁻¹ and 3430.74 cm⁻¹ are attributed to the bending and stretching vibrations, respectively, of O-H groups originating from water adsorbed molecules on the surfaces of the tested materials^50,51^. Moreover, the noticed absorption band at 1026.91 cm⁻¹ can be ascribed to the asymmetric stretching vibration of Si-O-Si bonds within RG and RT samples^51^ while the band at 673.04 cm⁻¹ affiliates to Mg-O vibrations of the RT^52^. The distinctive vibrational modes corresponding to Al–O bonds were detected at 438.72 cm⁻¹ in both the RG and RT samples^53^. Regarding OSW-AC, the prominent absorption band at 2920.66 cm⁻¹ corresponds to aliphatic C–H stretching vibrations of CH, CH₂, and CH₃ moieties, while the peak at 2859.92 cm⁻¹ is characteristic of symmetric CH₂ stretching, and the spectral feature at 2345 cm⁻¹ indicates the presence of C ≡ C functional groups^31^. Moreover, the absorption band at 1435.74 cm⁻¹ is assigned to both asymmetric and symmetric C-H bending vibrations, while spectral features between 976.66 and 1380.78 cm⁻¹ likely originate from C-O group vibrations, and the band observed at 883.24 cm⁻¹ corresponds to out-of-plane C-H stretching modes^54,55^. The development of the OP-GPHC binder induced a distinct spectral shift in the Si–O–Si stretching vibration from 1014.37 cm⁻¹ to 1115.62 cm⁻¹, indicative of structural reorganization during the geopolymerization reaction. This transition arises from the formation of aluminum silicate hydrate phases (N-A-S-H, C-A-S-H, and M-A-S-H), characterized by Si–O–T (T = Si/Al) asymmetric stretching modes. Furthermore, FTIR analysis revealed the emergence of a new absorption band at 3781.72 cm⁻¹, assigned to structurally incorporated water molecules within the created hydration products^56^, along with enhanced O-H stretching vibrations at 1638.23 cm⁻¹ (H-O-H bending) and 3430.74 cm⁻¹ (hydrogen-bonded hydroxyl groups). Furthermore, an additional absorption band was determined at 614.22 cm⁻¹, corresponding to Fe-O vibrational modes characteristic of both magnetite and magnesioferrite phases^57^.

**Fig. 3:**
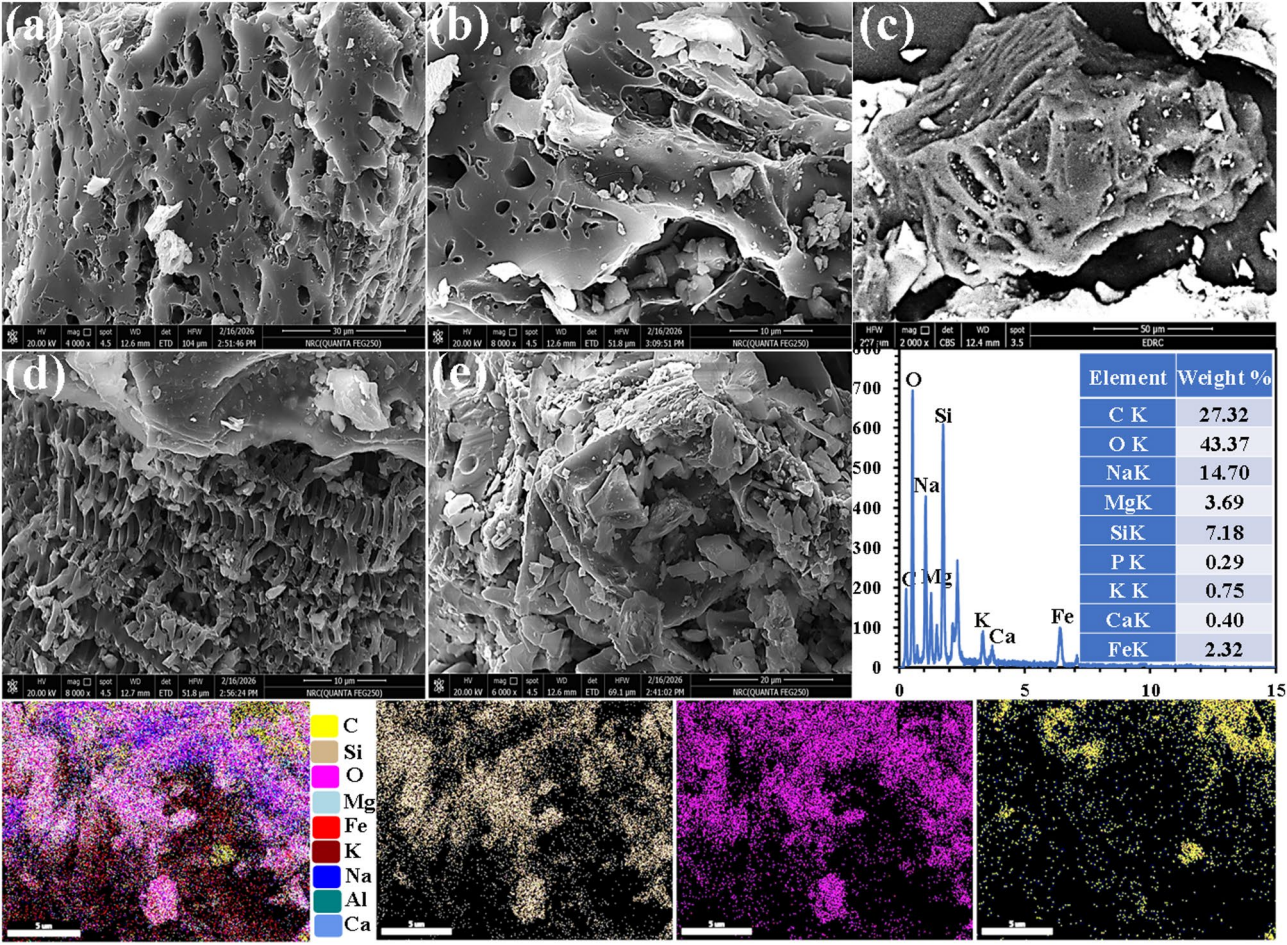
FESEM images (**a**–**d**), FESEM/EDX and elemental mapping analysis (**e**) of OP-GPHC adsorbent.

The N₂ adsorption-desorption isotherm data obtained for the fabricated OP-GPHC (as illustrated in Fig. 2c) yield critical information regarding its textural attributes and porous architecture. The isotherm conforms to a Type IV profile with an accompanying H3 hysteresis loop, as defined by IUPAC nomenclature, thereby substantiating the existence of a hierarchically organized pore system. This configuration is indicative of a composite framework characterized by the coexistence of mesoporous and macroporous features, along with a minor contribution from micropores. Quantitative assessment via N₂ physisorption reveals that the OP-GPHC adsorbent exhibits a substantial specific surface area of 3.8067 m²/g and an average pore diameter of 20.142 nm. The resultant porosity, coupled with the well-developed meso-macroporous network, plays a pivotal role in augmenting molecular accessibility. Such structural attributes facilitate enhanced adsorption kinetics and improved uptake capacity for CR by promoting more effective interfacial interactions between the adsorbent surface and the adsorbate molecules. Furthermore, the synthesized adsorbent exhibits a pore volume of 0.0192 cm³/g, a characteristic that facilitates the unimpeded mass transport of target contaminants and consequently augments the overall efficacy of CR sequestration. The remarkable adsorptive performance of OP-GPHC, however, cannot be attributed solely to its physical surface characteristics; rather, it is fundamentally underpinned by its pronounced chemical functionality. This functionality arises from a synergistic integration of diverse active sites conducive to chemical bonding such as π-π stacking interactions, which are particularly advantageous for the adsorption of aromatic pollutants. Concurrently, the geopolymeric matrix, in conjunction with OSW-AC, constitutes the principal adsorptive component. This composite not only supplies reactive sites and a layered structural configuration but also incorporates surface hydroxyl groups. These groups fulfill a dual role, providing an essential physical scaffold while concurrently contributing primary active sites, thereby collectively enhancing the material’s capacity for the efficient removal of CR. The development of a porous architecture within OP-GPHC is attributable to its markedly irregular surface topography (Fig. 3), which facilitates the formation of a complex, interconnected network of mesopores and macropores exhibiting a multimodal size distribution. This observed textural morphology aligns closely with the FESEM microstructural evidence. Furthermore, quantitative assessment through subsequent adsorption isotherm studies establishes a definitive structure-function relationship, substantiated by the material’s outstanding sequestration capacity for CR dye.

TG/DTG analysis was employed to conduct a comprehensive quantitative and qualitative assessment of the phase evolution in OP-GPHC, as depicted in Fig. 2d. Upon subjecting the developed binder to controlled heating up to 1000 °C, three endothermic transitions were discernible. The first endothermic reaction, occurring between 50 and 200 °C, results from the dehydration and progressive thermal degradation of hydration products, primarily metastable C-A-S-H^52^, accompanied by a measurable mass loss of 5.73%. urthermore, the second DTG curve consistently demonstrates a distinct peak associated with the thermal decomposition of the M-A-S-H and N-A-S-H phases^46,58^. A substantial mass loss of 2.89% was recoded in the temperature range of 200–400 °C, indicative of the liberation of structural water molecules and subsequent dissociation of hydroxyl groups. The final endothermic transition, occurring above 600 °C and corresponding to a mass loss of 2.46%, is attrbuted to the decarbonation process^52^. Moreover, the observed 8.62% mass reductin observed across the 50–400 °C temperature range strongly confirms the existence of hydration products, particularly C-A-S-H, M-A-S-H, and N-A-S-H phases. This thermogravimetric behavior further demonstrates the composite’s enhanced thermal stability, as it maintains structural integrity at higher temperatures before undergoing complete decomposition.

The morphological features and microstructural arrangement of the OP-GPHC adsorbent were examined using FESEM/EDX and elemental mapping, as depicted in Fig. 3 under varying magnifications. Upon completion of the vitrification and geopolymerization processes, the resultant material exhibits a predominantly reactive amorphous microstructure, characterized by a porous glassy matrix (Fig. 3a,b). Microscopic analysis confirms this phase composition, revealing a complete absence of crystalline features that would otherwise indicate the presence of the original RG and RT constituents. Moreover, the OSW-AC exhibits a flaky, porous morphology (Fig. 3c) that provides an optimal framework for the rapid diffusion and adsorption of CR molecules. Figure 3d illustrates the presence of glassy phase layers serving as a covering material on the OSW-AC, highlighting the effective interfacial interaction between the two components. Further microstructural examination (Fig. 3e) reveals that the OP-GPHC adsorbent is characterized by an angular and flaky morphology, accompanied by a pronounced heterogeneity in particle dimensions. The phase assemblage is principally composed of glassy matrices interspersed with hydration products, specifically including C-A-S-H, M-A-S-H, and N-A-S-H. Additionally, the material incorporates magnetic mineral phases, notably magnetite and magnesioferrite. The resultant composite matrix exhibits a porous surface texture and contains particulates spanning a wide size distribution, features that collectively augment its capacity for CR sequestration. FESEM-EDX analysis coupled with elemental mapping was performed on OP-GPHC (Fig. 3e), providing definitive confirmation of both its elemental stoichiometry and the spatial distribution of its constituent components. The EDX spectra quantitatively substantiate the intended elemental composition, thereby affirming the successful fabrication of the target adsorbent. Corresponding elemental maps reveal silicon (light brown), carbon (yellow), and oxygen (purple) as the principal constituents, while magnesium (light blue), iron (red), potassium (dark red), sodium (blue), aluminum (dark cyan), and calcium (medium blue) are also detected as minor elemental components.

### Adsorption study of CR on OP-GPHC adsorbent

#### Effect of solution pH on CR removal efficiency

**Fig. 4:**
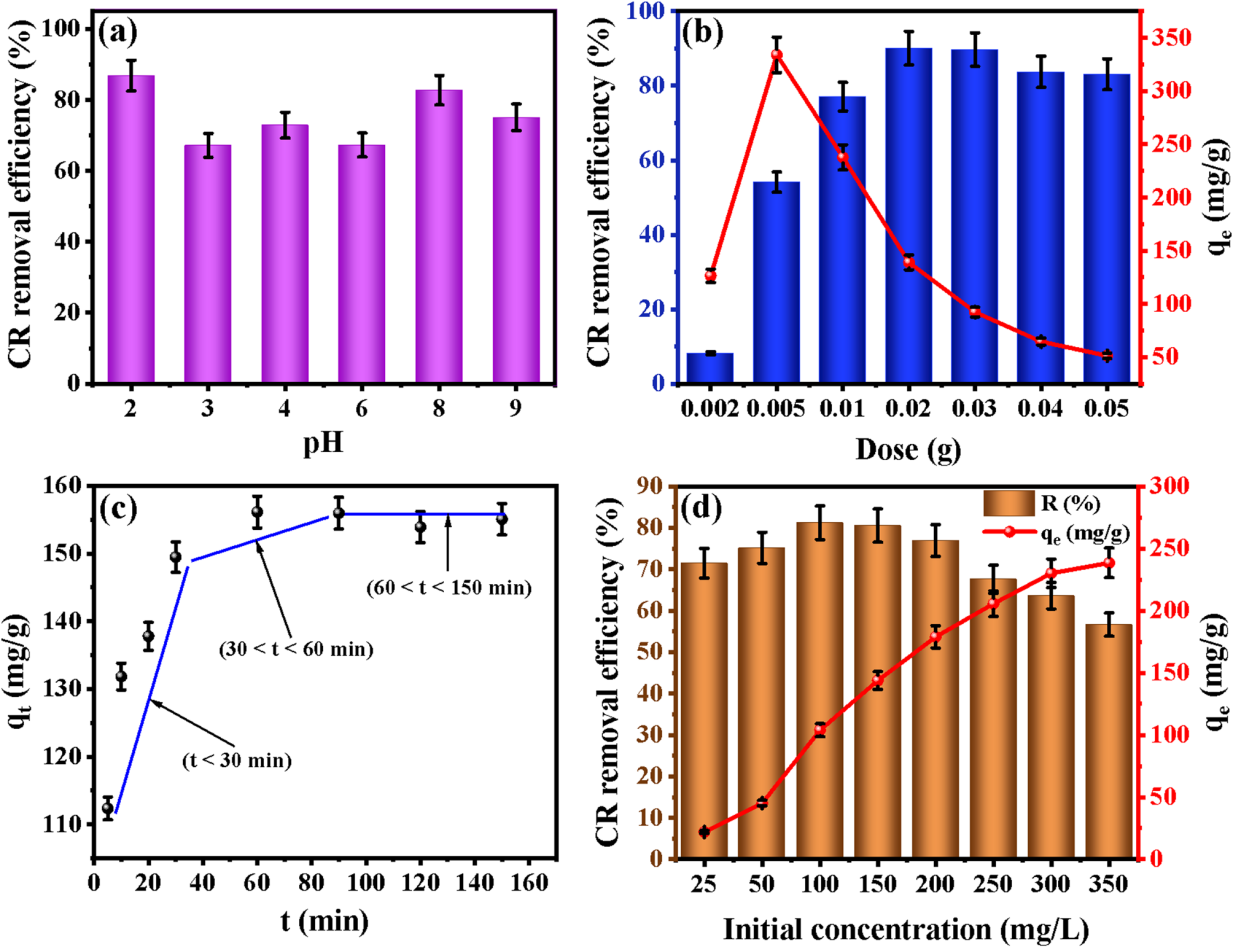
(**a**) Effect of solution pH [conditions: 0.02 g adsorbent, 25 mL of 150 mg/L CR solution, 60 min contact time, 25 °C], (**b**) Effect of OP-GPHC dosage [conditions: pH 2.0, 25 mL of 150 mg/L CR solution, 60 min contact time, 25 °C], (**c**) Effect of contact time [conditions: pH 2.0, 0.02 g adsorbent, 25 mL of 150 mg/L CR solution, 25 °C], and (**d**) Effect of initial CR concentration [conditions: pH 2.0, 0.02 g adsorbent, 25 mL solution, 60 min contact time, 25 °C].

The adsorption performance of CR onto the OP-GPHC remained robust across a wide initial pH range of 2 to 9, as illustrated in Fig. 4a. Maximum removal efficiency (99.2%) was achieved at pH 2. According to pH drift analysis (Fig. S1), the point of zero charge (pH_pzc_) of OP-GPHC was determined to be approximately 7. Below this value, the surface functional moieties, including C–O, –OH, Si–O–Si, Al–O, and Fe–O, undergo protonation, acquiring a net positive charge that establishes favorable electrostatic complementarity with the anionic sulfonate (SO₃⁻) groups of the CR dye. This electrostatic attraction constitutes the primary driving force behind the high adsorption capacity observed under strongly acidic conditions^59^.

Even at pH 8, where the adsorbent surface carries a net negative charge (as pH exceeds pH_pzc_), the removal efficiency remained above 80%. This sustained performance under alkaline conditions points to the involvement of non-electrostatic mechanisms. First, hydrogen bonding between surface hydroxyl groups (–OH) of OP-GPHC and the amino (–NH₂) or azo (–N = N–) functionalities of CR becomes predominant when electrostatic repulsion is operative. Second, π–π stacking interactions between the conjugated aromatic rings of CR and the graphitic domains of the olive seed waste–derived activated carbon component remain unaffected by pH variations and contribute meaningfully to dye retention. Third, the appreciable magnesium and iron content of the composite—originating from talc glauconite phases—enables surface complexation or inner-sphere coordination with sulfonate groups, a process that remains favorable even under mildly alkaline conditions. Collectively, these mechanisms explain the consistently high removal efficiency (> 80%) across a broad pH spectrum, with maximum uptake at pH 2 driven predominantly by electrostatic attraction^11,59^.

When the pH exceeded 9, a sharp decline in adsorption efficiency was observed. Under such highly alkaline conditions, an elevated concentration of hydroxide ions (OH⁻) competes directly with anionic dye molecules for the same binding sites, thereby suppressing overall dye uptake. Conversely, at pH 2—well below the pHpzc—surface aluminol (Al–OH) and silanol (Si–OH) groups become protonated to form Al–OH₂⁺ and Si–OH₂⁺ species. This protonation-induced surface charge reversal creates a strongly positively charged interface, resulting in powerful electrostatic attraction toward the anionic CR dye. This mechanism explains the superior adsorption performance recorded under strongly acidic conditions^3,12^.

#### Role of OP-GPHC dosage

By employing varying adsorbent doses ranging from 0.002 to 0.05 g/25 ml, researchers successfully assessed the influence of OP-GPHC dosage on the adsorption efficiency of CR molecules. As depicted in Fig. 4b, an increase in adsorbent mass resulted in enhanced removal of CR, attributable to the greater availability of active surface sites. A maximum removal efficiency of 90.09% was achieved at a low adsorbent dosage of 0.02 g, underscoring the exceptional adsorption capacity of the adsorbent material. Beyond the optimal dosage, the removal efficiency stabilizes, indicating that further increases in adsorbent quantity do not improve pollutant adsorption^60^. Excessive adsorbent use leads to particle aggregation^61^, reducing the availability of active sites and diminishing overall removal performance. The existence of abundant reactive sites in the inorganic (geopolymer) and organic (activated carbon) components enables superior adsorption capacity, even at a low adsorbent dosage (0.02 g). This demonstrates a strong synergistic interaction between the two constituent materials forming the developed binder.

#### Influence of interaction time

Analysis of the adsorption kinetics for CR on the OP-GPHC material, presented in Fig. 4c, reveals a triphasic uptake mechanism. The kinetic analysis unequivocally demonstrates three well-defined adsorption regimes for CR: an instantaneous rapid uptake phase (t < 30 min), a diffusion-controlled intermediate phase (30 < t < 60 min), and a near-equilibrium saturation phase (60 < t < 150 min). The exceptionally high initial adsorption rate, attributed to the overwhelming abundance of unoccupied functional groups on the OP-GPHC binder, underscores its superior affinity for CR dye. The intermediate adsorption phase exhibits intra-particle diffusion-limited mass transfer, resulting in markedly reduced CR uptake. During the final equilibrium stage (≤ 150 min), the adsorbed pollutant concentration stabilized, indicating surface saturation and achievement of dynamic equilibrium, where further contaminant retention became thermodynamically unfavorable.

#### Impact of CR initial feed concentration

The adsorption of CR by the OP-GPHC composite is strongly influenced by its initial concentration (Fig. 4d). Testing across 25–350 mg/L reveals two complementary trends. Removal efficiency inversely correlates with concentration, dropping from 97.8% at 25 mg/L to 56.64% at 350 mg/L. This reflects the finite active sites on OP-GPHC: at low concentrations, the site-to-molecule ratio is high, enabling near-complete removal, while higher concentrations lead to site saturation and reduced efficiency, consistent with mass action principles^51^. Conversely, adsorption capacity (q_e_) increases with concentration, rising from 150.9 mg/g at 25 mg/L to 253.5 mg/g at 350 mg/L. This is driven by an enhanced concentration gradient that facilitates mass transfer. The q_e_ rises sharply at lower concentrations (25–100 mg/L), moderates at intermediate levels (100–250 mg/L) as sites fill, and plateaus at 250–350 mg/L, signaling site saturation, defining the material’s maximum adsorption capacity under these conditions^50,57^. Together, these metrics offer a holistic view: removal efficiency reflects practical treatment performance, while adsorption capacity reveals intrinsic material potential. OP-GPHC combines high removal efficiency at environmentally relevant concentrations (> 80% at 150 mg/L) with exceptional capacity (253.5 mg/g at 350 mg/L), highlighting its suitability for treating CR-contaminated wastewater across diverse concentration scenarios.

### BBD optimization for CR adsorption process

#### Statistical investigations

Analysis of variance (ANOVA) serves as a pivotal statistical tool for evaluating model accuracy, rigidity, and the association between independent variables and their corresponding outputs, as presented in Table 3. Furthermore, Table 4 demonstrates that the quadratic model exhibited the best fit for CR adsorption data among all evaluated models. To evaluate the adequacy of the quadratic model in describing the experimental results, the statistical significance was assessed by analyzing the p-value and *F*-value derived from the ANOVA outcomes, as summarized in Table 5 for CR removal. A model variable demonstrating an elevated *F*-value in conjunction with a p-value below the 0.05 threshold was measured statistically significant^11^. The *F*-values for the majority of the models demonstrate substantially higher magnitudes relative to their corresponding p-values, with most model *p*-values falling below the 0.05 threshold. Moreover, a satisfactory correlation was observed between the adjusted R² (0.98) and predicted R² (0.87) values for CR removal, indicating the model’s predictive accuracy^11^. Based on the ANOVA results, this indicates that the proposed model is statistically significant and exhibits a robust relationship between its predictive capability and the independent variables.

The optimization of CR adsorption can be effectively investigated through a quadratic regression model. The statistically significant parameters in this model include: = A, B, C, along with their binary interaction terms (AB, AC, BC) and quadratic terms (A², B², C²). The empirical correlation between the adsorption performance of CR and the investigated parameters was mathematically modeled, as signified by Eq. (4) for CR adsorption.

4 $$\begin{aligned} {\text{Removal }}percentage{\text{ }}\left( \% \right){\text{ }} & = {\text{ }}86.74{\text{ }} - {\text{ }}35.49{\text{ }}x{\text{ }}A{\text{ }} + {\text{ }}13.18{\text{ }}x{\text{ }}B{\text{ }} + {\text{ }}15.75{\text{ }}x{\text{ }}C{\text{ }}{-}{\text{ }}0.4025{\text{ }}x{\text{ }}AB{\text{ }} \\ & {-}{\text{ }}5.75{\text{ }}x{\text{ }}AC{\text{ }} + {\text{ }}4.79{\text{ }}x{\text{ }}BC{-}{\text{ }}10.89{\text{ }}x{\text{ }}A^{2} {-}{\text{ }}25.76{\text{ }}x{\text{ }}B^{2} {-}{\text{ }}30.80{\text{ }}x{\text{ }}C^{2} \\ \end{aligned}$$

The regression equations expressed in coded factors enable the prediction of system responses at specified levels of each variable. In this coding scheme, factor levels are standardized, with + 1 representing the high level and − 1 denoting the low level. The relative significance of individual factors can be assessed by comparing their standardized coefficients within the coded model^50^. Generally, the ANOVA results collectively demonstrate that the advanced regression models effectually characterize the relationship between the independent variables and CR removal, exhibiting statistically significant accuracy and reliability^62^.

**Table 3:** The adsorption capacity of CR, along with the corresponding RSM results of the central composite design.

Run order	Variables			Response (removal %)	
	pH	Adsorbent dose (g)	Contact time (min)	CR	
				Actual	Predicted
1	5.5	0.01	150	32.63	27.96
2	5.5	0.07	150	68.35	63.89
3	5.5	0.07	5	18.14	22.81
4	5.5	0.04	77.5	86.74	86.74
5	2	0.07	77.5	100	99.16
6	5.5	0.04	77.5	86.74	86.74
7	9	0.07	77.5	26.74	27.37
8	9	0.04	150	15.74	19.57
9	5.5	0.04	77.5	86.74	86.74
10	2	0.04	150	96.74	102.04
11	9	0.01	77.5	0.98	1.82
12	2	0.04	5	62.87	59.04
13	5.5	0.04	77.5	86.74	86.74
14	2	0.01	77.5	72.63	72
15	5.5	0.04	77.5	86.74	86.74
16	5.5	0.01	5	1.58	6.04
17	9	0.04	5	4.86	-0.4413

**Table 4:** Statistical analysis of diverse CR adsorption models.

Source	Sum of squares	df	Mean square	Std. dev.	Adjusted *R*^2^	Predicted *R*^2^	Remark
Linear	25.4	0.6158	0.5272	0.4183	0.5272	0.4183	
2FI	28.58	0.6261	0.4018	0.0038	0.4018	0.0038	
Quadratic	4.94	0.9922	0.9821	0.8747	0.9821	0.8747	Suggested
Cubic	0	1	1		1		Aliased

**Table 5:** Analysis of the fitted models’ variance.

Source	Sum of squares	df	Mean square	F-value	*p*-value	Significance
Model	21669.21	9	2407.69	98.52	< 0.0001	Significant
A	10076.32	1	10076.32	412.33	< 0.0001	Significant
B	1388.91	1	1388.91	56.83	0.0001	Significant
C	1984.82	1	1984.82	81.22	< 0.0001	Significant
AB	0.648	1	0.648	0.0265	0.8752	Not significant
AC	132.14	1	132.14	5.41	0.053	Not significant
CB	91.78	1	91.78	3.76	0.0938	Not significant
A^2^	499.11	1	499.11	20.42	0.0027	Significant
B^2^	2795.1	1	2795.1	114.38	< 0.0001	Significant
C^2^	3994.27	1	3994.27	163.45	< 0.0001	Significant
Residual	171.06	7	24.44			
Lack of fit	171.06	3	57.02			
Pure error	0	4	0			
Cor total	21840.27	16				

#### Optimization of CR adsorption via 3D graphs representation

**Fig. 5:**
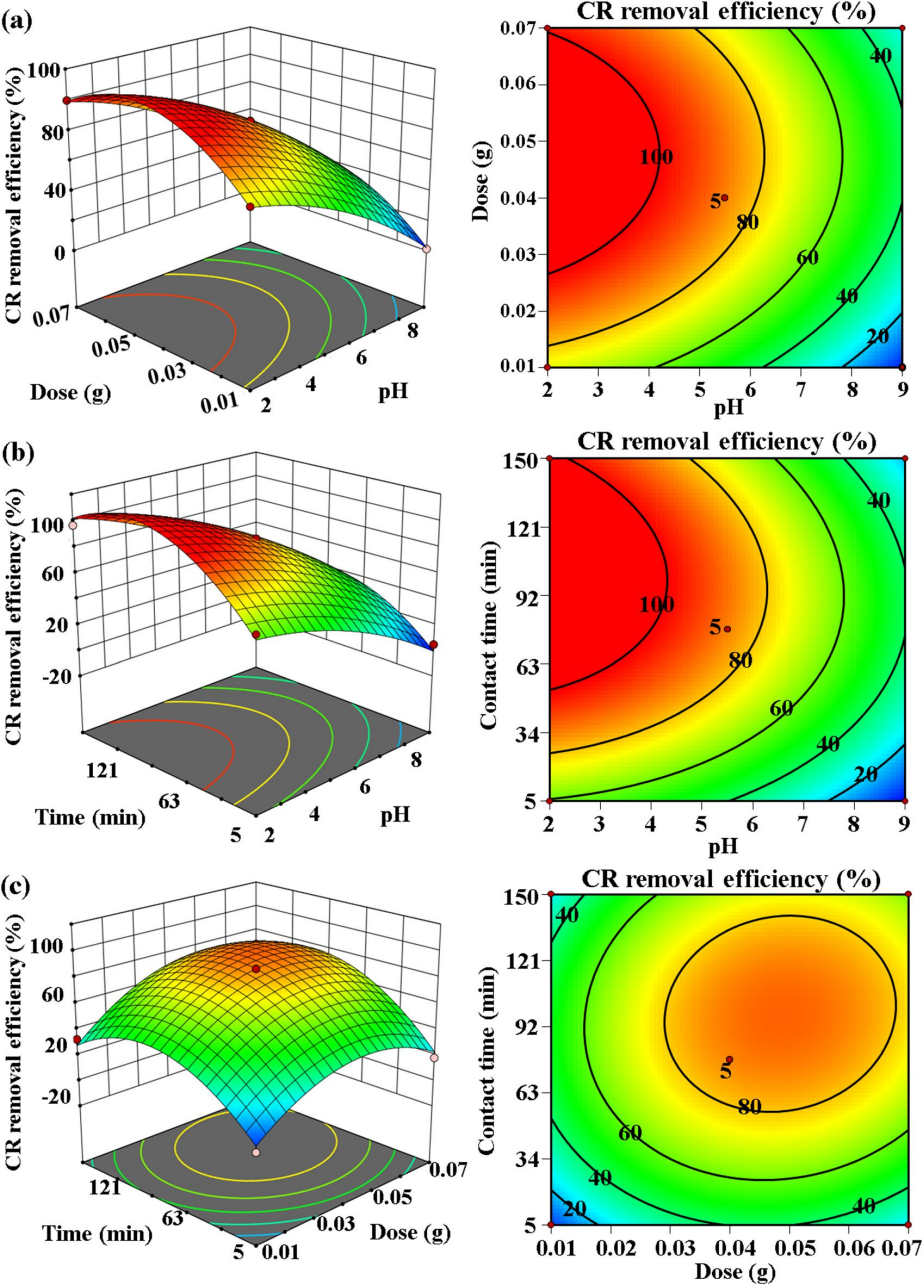
3D RSM graphs and contour plots illustrating significant interactive effects on CR removal efficiency for (**a**) AB, (**b**) AC, and (**c**) BC variable pairs.

To comprehensively investigate the synergistic interactions between individual components and rigorously optimize the adsorption parameters for maximal CR removal efficiency, the three-dimensional response surfaces were analytically evaluated as a function of the two critical independent variables. The 3D surface plots presented in Fig. 5 illustrate significant synergistic relationships between key operational parameters: (a) OP-GPHC dosage and solution pH, (b) contact duration and solution pH, and (c) contact duration and OP-GPHC dosage. Figure 5a demonstrates that the adsorption capacity of CR improves significantly with raising OP-GPHC dosage (0.01–0.07 g), exhibiting optimal removal at pH 2. This behavior arises from electrostatic interactions between the anionic CR molecules and the protonated OP-GPHC surface. Moreover, Fig. 5b illustrates a significant synergistic interaction between contact time and solution pH. This interaction leads to a marked growth in the adsorption efficiency of CR, with optimal exposure times of 150 min. This trend is attributed to extended interaction periods, which facilitate greater binding between OP-GPHC active sites and the target contaminant (CR dye), thereby maximizing adsorption capacity. The findings presented in Fig. 5c indicate a robust interaction between the OP-GPHC dosage and the contact time. This interaction significantly enhances the adsorption efficiency of CR as the OP-GPHC respective levels increase. The observed improvement can be accredited to the augmentation of available active sites within the OP-GPHC binder, which facilitates the adsorption of CR while providing adequate interaction time for optimal contact between the contaminant and the adsorbent, thereby demonstrating high removal efficiency.

#### Quadratic model suitability examination

**Fig. 6:**
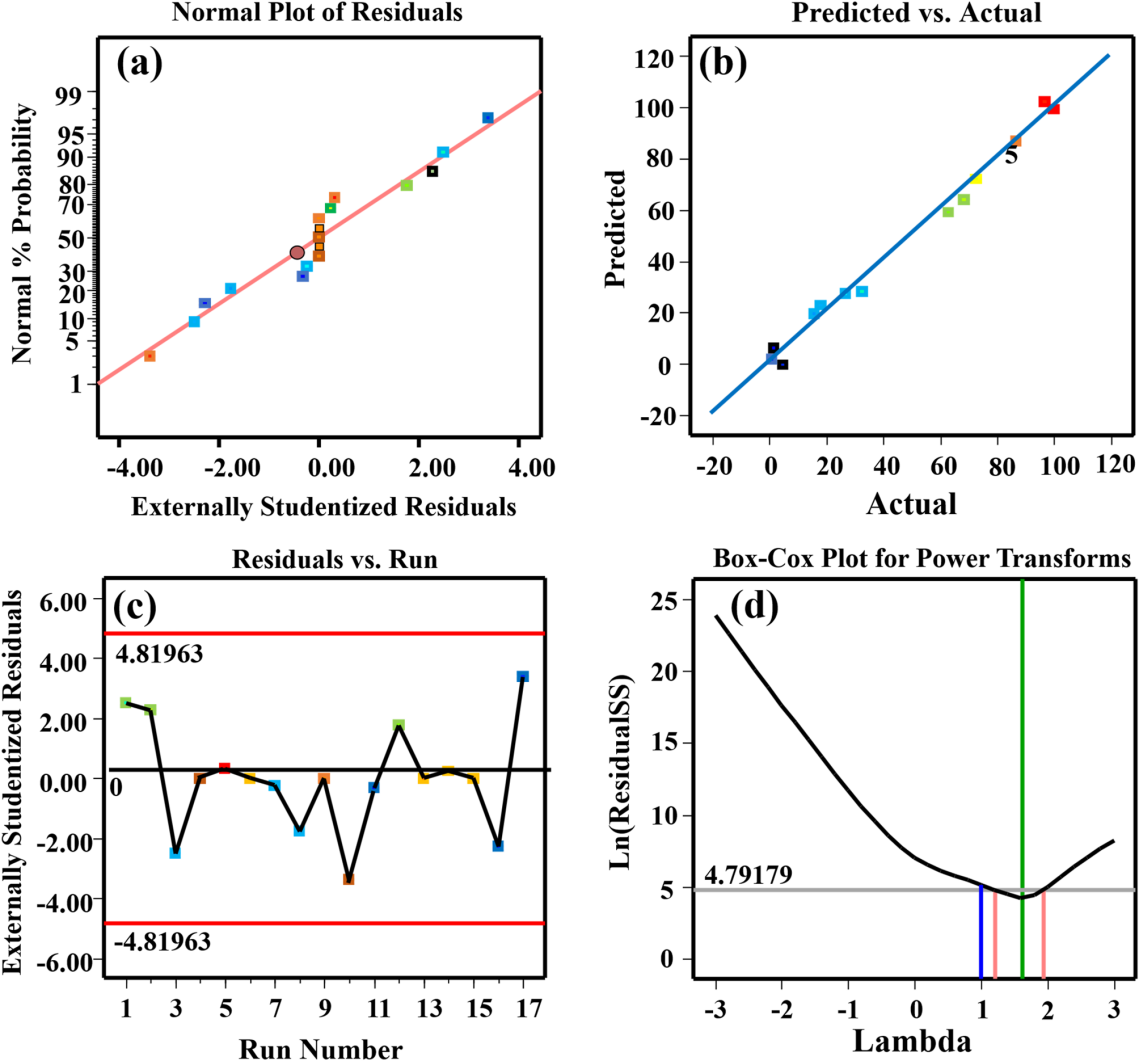
Diagnostic and optimization analyses for CR adsorption on OP-GPHC adsorbent: (**a**) normal probability plots of residuals, (**b**) actual versus predicted CR removal efficiency values, (**c**) residual distribution across runs, (**d**) Box-Cox transformation plots.

To validate the BBD model, the residual distribution and correlation between experimental and predicted values of CR adsorption capacity were analyzed graphically. Figure 6a presents the normal probability plot of residuals for the BBD model^51,62^, where the linear distribution pattern confirms both normal residual distribution and optimal independence. Figure 6b illustrates the correlation between model-predicted and experimentally determined adsorption capacities for CR. The close alignment of data points along the regression line demonstrates strong agreement between predicted and observed values, validating the model’s accuracy. The residual distributions plotted against experimental run sequences for CR adsorption processes are presented in Fig. 6c. These plots demonstrate randomly scattered residuals symmetrically distributed about the zero-reference line, indicating the absence of heteroscedasticity and thereby validating the statistical reliability of the developed predictive models. The Box-Cox transformation plots presented in Fig. 6d demonstrate the relationship between residual variance and lambda values for CR removal. Statistical analysis identifies optimal Box-Cox transformation parameters of lambda = 4.79, corresponding to maximum variance stabilization. The unity transformation parameter (lambda = 1)^57^ applied in the current model confirms that no power transformation was necessary for the CR removal dataset, preserving the original scale of measurement.

#### Perturbation plot and cubic interaction

The perturbation plot illustrates the relationships between all variables within the meticulously designed experimental space. As shown in Fig. S2a, this plot evaluates the adsorption capacity of CR onto OP-GPHC, examining the individual influence of key parameters (time, adsorbent dosage, and pH) while maintaining the remaining factors at constant levels. This plot serves as a crucial tool for assessing the sensitivity of the adsorption process to variations in individual parameters across their defined operational ranges. The data demonstrate how each factor significantly influences adsorption capacity at distinct points within the experimental domain, highlighting the efficacy of targeted parameter adjustments in optimizing process performance. The perturbation analysis reveals that each examined parameter (time, adsorbent dosage, and pH) exerts a favorable effect on adsorption capacity. Systematic modification of these variables may enhance overall system performance, thereby providing critical insights for process optimization and facilitating the achievement of maximum adsorption efficiency^50^. Fig. S2a demonstrates pronounced curvature effects induced by variations in contact time, pH, and adsorbent dosage. The adsorption capacity exhibits rapid responsiveness to these operational parameters, particularly revealing a strong correlation between solution pH and adsorption duration^62^. Moreover, the effects of the analyzed variables (i.e., pH, OP-GPHC dosage, and contact time) on the cubic RSM graphs were presented in Fig. S2b. The plot elucidates the interplay between adsorbent dose and contact time in the removal of CR, underscoring divergent adsorption mechanisms of OP-GPHC for these pollutants in aqueous media. For CR dye, adsorption efficiency exhibits a positive correlation with extended contact duration. Through systematic optimization of experimental parameters, quantitative contaminant removal (99.16% ± 5%) was achieved under the following conditions: 0.07 g adsorbent dosage, pH 2, and a contact time of 77.5 min^57^. Subsequent experimental iterations employed a further optimized adsorbent mass of 0.02 g to maximize adsorption capacity (*q*_*e*_) while maintaining optimal active site utilization efficiency.

#### The desirability method and model validation

**Fig. 7:**
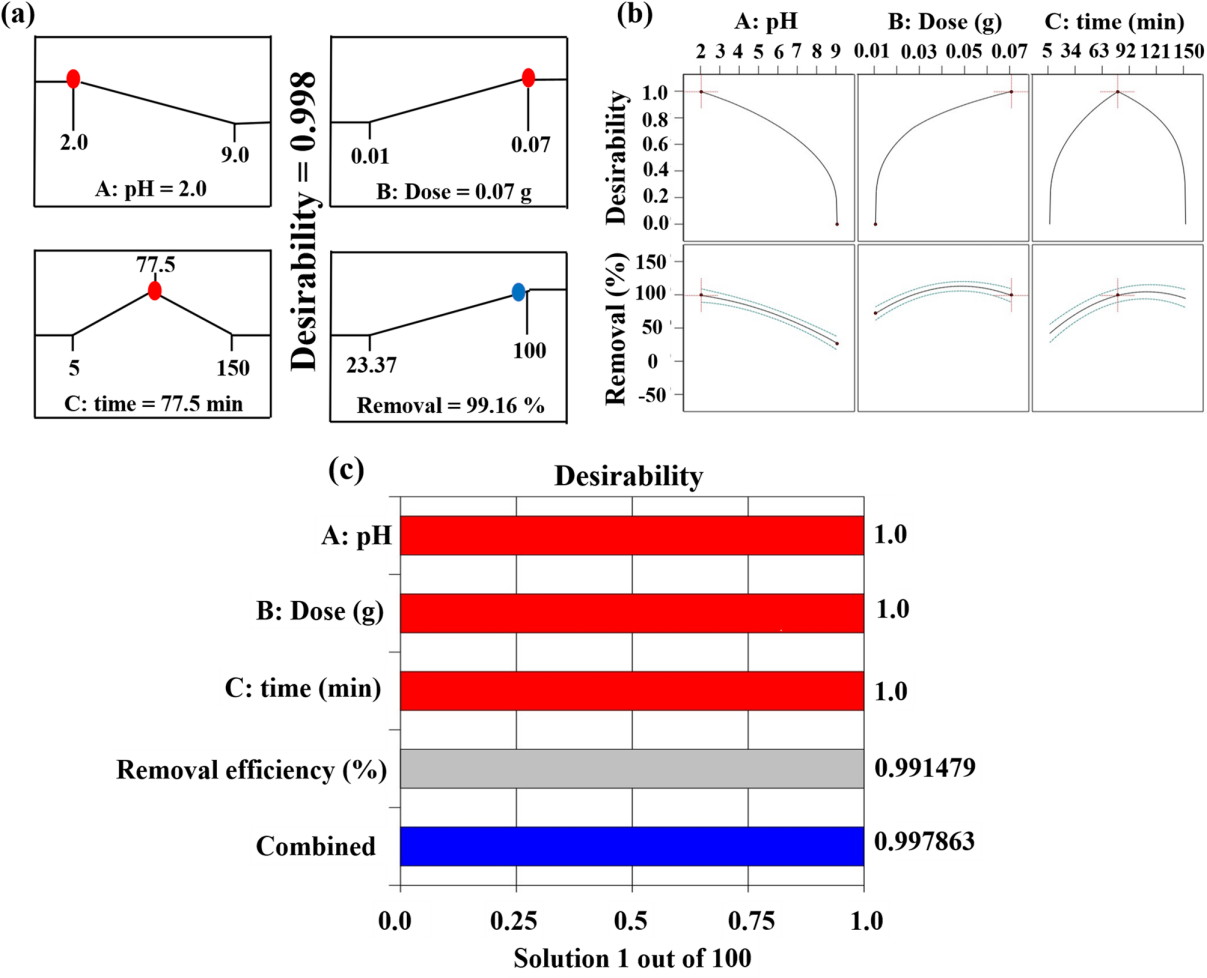
(**a**) Rising focus on optimal numerical solutions, (**b**) response desirability of each factor, and (**c**) individual desirability as a bar chart.

The desirability function approach was employed to optimize the adsorption capacity of CR, achieving maximal removal efficiency while simultaneously minimizing resource requirements. This multi-objective optimization balanced production goals with operational constraints, maintaining adsorption performance without increasing chemical inputs, energy consumption, or labor demands. The optimization analysis identified optimal operational parameters that maximize adsorption performance^63^. Accordingly, Fig. 7a quantifies the ramp appeal exhibited by the optimized solution, which was rigorously identified through computational numerical optimization. The process optimization study identified pH 2, a contact time of 77.5 min, and an adsorbent mass of 0.07 g as the optimal operational parameters for achieving high CR removal efficiency, with a maximum removal rate of 99.16% (Fig. 7b). The optimization conditions (pH 2, a contact time of 77.5 min, and an adsorbent mass of 0.07 g) were validated experimentally, yielding a complete removal efficiency of 100%, s documented in Table 5 on page 29. The model’s predicted value of 99.16% i therefore highly consistent with the experimental result. These findings are substantiated by the desirability analysis presented in Fig. 7b, where all normalized response values conform to the standardized 0–1 desirability scale. Within this framework, a value of 0 denotes completely unsatisfactory conditions, while unity (1.0) signifies the theoretically ideal response. Figure 7c presents a graphical representation of individual desirability functions, employing bar plots to illustrate the relationship between input variables and response parameters for CR removal efficiency^63^. The optimized input parameters were subsequently validated through two independent verification trials. The numerical optimization outcomes derived from desirability function analysis demonstrate strong concordance with the experimental results. The optimization study established that the BBD coupled with desirability function analysis effectively identified optimal adsorption conditions for maximum removal efficiency. Figure 7b present the validated experimental parameters that yield peak adsorption performance in this investigation.

### Adsorption kinetics and diffusion mechanism for CR adsorption

**Fig. 8:**
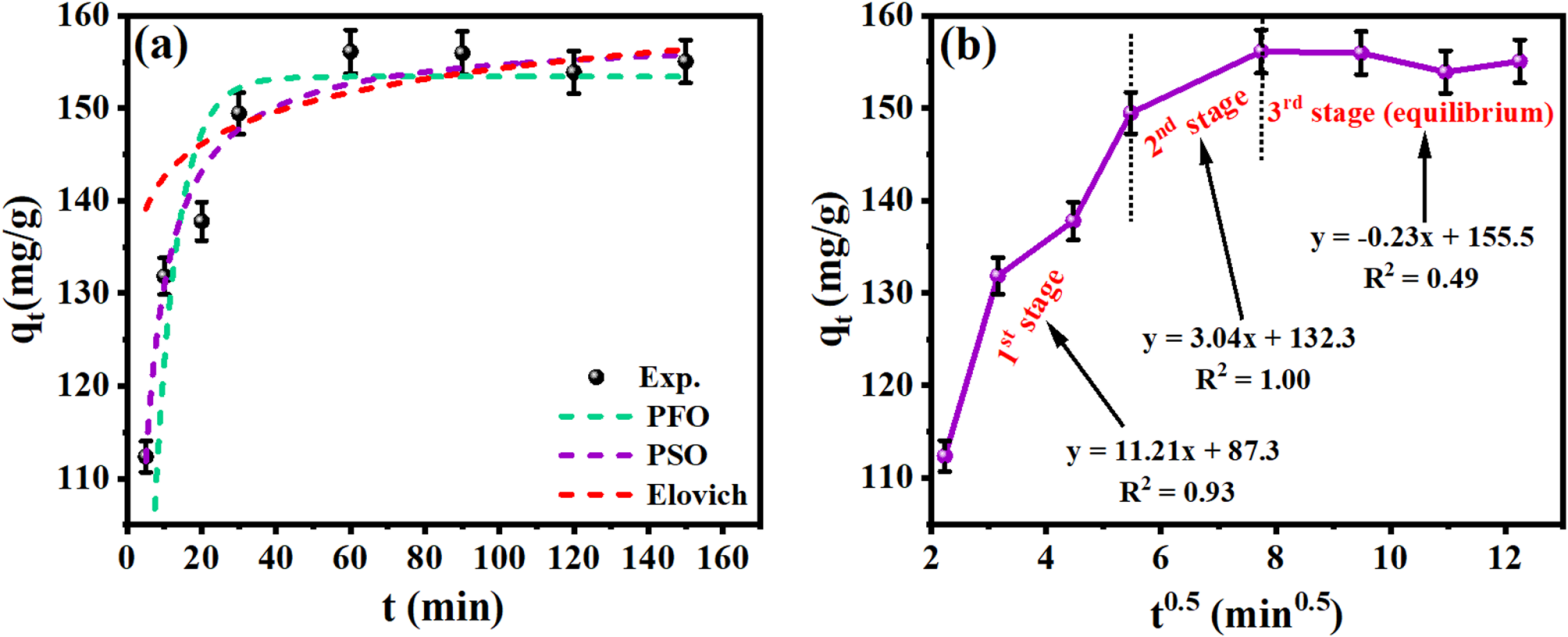
Kinetic modeling of CR uptake on OP-GPHC: (**a**) nonlinear fits of the PFO, PSO, and Elovich models; and (**b**) the IPD plot.

The kinetic behavior characterizing the adsorption of CR molecules onto the OP-GPHC surface was evaluated through a suite of models, including pseudo-first-order (PFO), pseudo-second-order (PSO), Elovich, and intraparticle diffusion (IPD). This integrated modeling strategy serves a dual purpose: it deciphers the fundamental adsorption mechanism and supplies a data-driven basis for the optimization of process conditions to maximize the efficacy of contaminant sequestration. The kinetic parameters for the PFO^3,32^ and PSO^34,50,51^ models were derived for CR adsorption through the analysis of nonlinear regression plots depicting $\:{q}_{t}$ related to time, as shown in Fig. 8a. The specific numerical values associated with these parameters are comprehensively outlined in Table 6.

Compared to the PSO model, the PFO equation demonstrated a superior fit to the CR adsorption data, as indicated by the higher $\:{R}^{2}$ values. Moreover, the PFO model showed strong agreement with the experimental data, as the calculated $\:{q}_{e}$ values closely corresponded to the experimentally observed $\:{q}_{t}$ values. The exceptional conformity of the kinetic data to the Elovich model ($\:{R}^{2}=0.995$; Fig. 8a) provides definitive evidence that chemisorption also governs the adsorption of CR onto the OP-GPHC composite. This high correlation coefficient signifies a process characterized by a heterogeneous surface with an exponential decrease in adsorption energy as sites are occupied. The derived Elovich parameters, $\:\alpha\:$ (8.087 × 10¹¹) and $\:\beta\:$ (0.197 × 10⁻⁴) detailed in Table 6, quantitatively support this; a high $\:\alpha\:$ value reflects a rapid initial uptake, while a substantial $\:\beta\:$ parameter indicates strong binding forces between the composite’s functional groups and the dye molecules, likely through charge-assisted coordination or complexation^51,57^.

Critically, the concurrent validity of both the Elovich and PFO models necessitates a more nuanced interpretation than either model offers alone. While PFO is conventionally associated with physisorption and Elovich with chemisorption, their simultaneous good fit is not contradictory but rather reflects a composite adsorption mechanism arising from the heterogeneous nature of the OP-GPHC surface. In this framework, the initial rapid phase (first 60 min) is dominated by chemisorptive interactions—such as hydrogen bonding, $\:\pi\:$-$\:\pi\:$ stacking, and electrostatic complexation with surface functional groups—which are well-described by the Elovich equation. Subsequently, as the high-affinity sites become saturated, the approach to equilibrium is governed by complementary physisorptive processes, including pore diffusion, van der Waals interactions, and weaker electrostatic attractions, which the PFO model partially captures. Thus, rather than indicating a single rate-limiting step, the concurrent model validity reveals a heterogeneous surface chemistry where multiple mechanisms operate across different time scales.

The diagnosis of rate-controlling phenomena is greatly enhanced by a multi-model interrogation. The IPD model is essential to delineate boundary layer effects from intra-particle transport, while the PSO model offers a critical counterpoint; its comparatively lower fit in this context further reinforces the conclusion that chemical valence forces, rather than mere mass transfer or sharing of electrons, are the dominant driver during the initial uptake stage. This consolidated analytical approach not only deciphers the fundamental mechanism but also provides a robust empirical basis for optimizing contact time and scaling up the process for efficient contaminant removal^50^.

The application of the IPD model to the kinetic data reveals a multi-stage mass transport process for CR sequestration onto the OP-GPHC adsorbent. The resultant $\:{q}_{t}$ versus $\:{t}^{0.5}$ plot (Fig. 8b) exhibits a distinct triphasic linear profile, signifying that the adsorption is governed by a sequence of distinct mechanistic steps rather than a single, uniform process. The initial, steep linear segment corresponds to the rapid migration of CR molecules across the boundary layer and their instantaneous attachment to readily accessible sites on the adsorbent’s exterior surface. This film diffusion stage, characterized by a relatively high-rate constant of 11.21 mg/g·min¹/²^64^, is evidenced by the plot’s significant y-intercept, confirming it as a primary and rapid initial driving force. The subsequent, more gradual linear phase signifies the onset of intra-particle diffusion, where CR molecules migrate into the adsorbent’s porous structure. The significantly reduced rate constant for this stage (3.04 mg/g·min¹/²) reflects the increased mass transfer resistance inherent to diffusion within the constrained geometric architecture of the material’s internal pore network. The final plateau phase, where the diffusion rate approaches zero, denotes the establishment of dynamic equilibrium. At this stage, the driving force for adsorption diminishes due to both the saturation of available binding sites and the reduction in the solute concentration gradient. The multi-linear nature of the kinetic profile provides conclusive evidence that the adsorption process is governed by two concurrent mechanisms: rapid surface film diffusion and a slower, rate-limiting intra-particle transport^35^.

**Table 6:** Performed kinetic parameters to assess of CR uptake via OP-GPHC.

Model		Variable		Unit			Value		Std. error		t- value		p‑value†	
PFO		*q*_*e*_		mg/g			153.4		2.5		61.37		1.258×10^-9^	
		*K*_*1*_		min^-1^			0.161		0.03		6.256		7.739×10^-4^	
		*R* ^*2*^					0.998							
		*X* ^*2*^					5.631							
PSO		*q*_*e*_		mg/g			157.88		1.56		101.18		6.282×10^-11^	
		*K*_*2*_		g/mg.min			0.003		2.64×10^-4^		11.69		2.365×10^-5^	
		*R* ^*2*^					0.977							
		*X* ^*2*^					1.792							
Elovich		*α*		mg/g.min			8.087×10¹¹		0		-		-	
		*β*		g/mg			0.197×10^-4^		1.324×10^-4^		1487.95		0	
		*R* ^*2*^					0.995							
		*X* ^*2*^					9.194							

Intraparticle diffusion (IPD) model								
Step 1			Step 2			Step 3		
*K*_*P*_1	*C*_*1*_	*R* ^*2*^	*K*_*P*_2	*C*_*2*_	*R* ^*2*^	*K*_*P*_3	*C*_*3*_	*R* ^*2*^
11.21	87.3	0.93	3.04	132.3	1	~0	155.5	0.49

### Traditional adsorption isotherms evaluation for CR removal

**Fig. 9:**
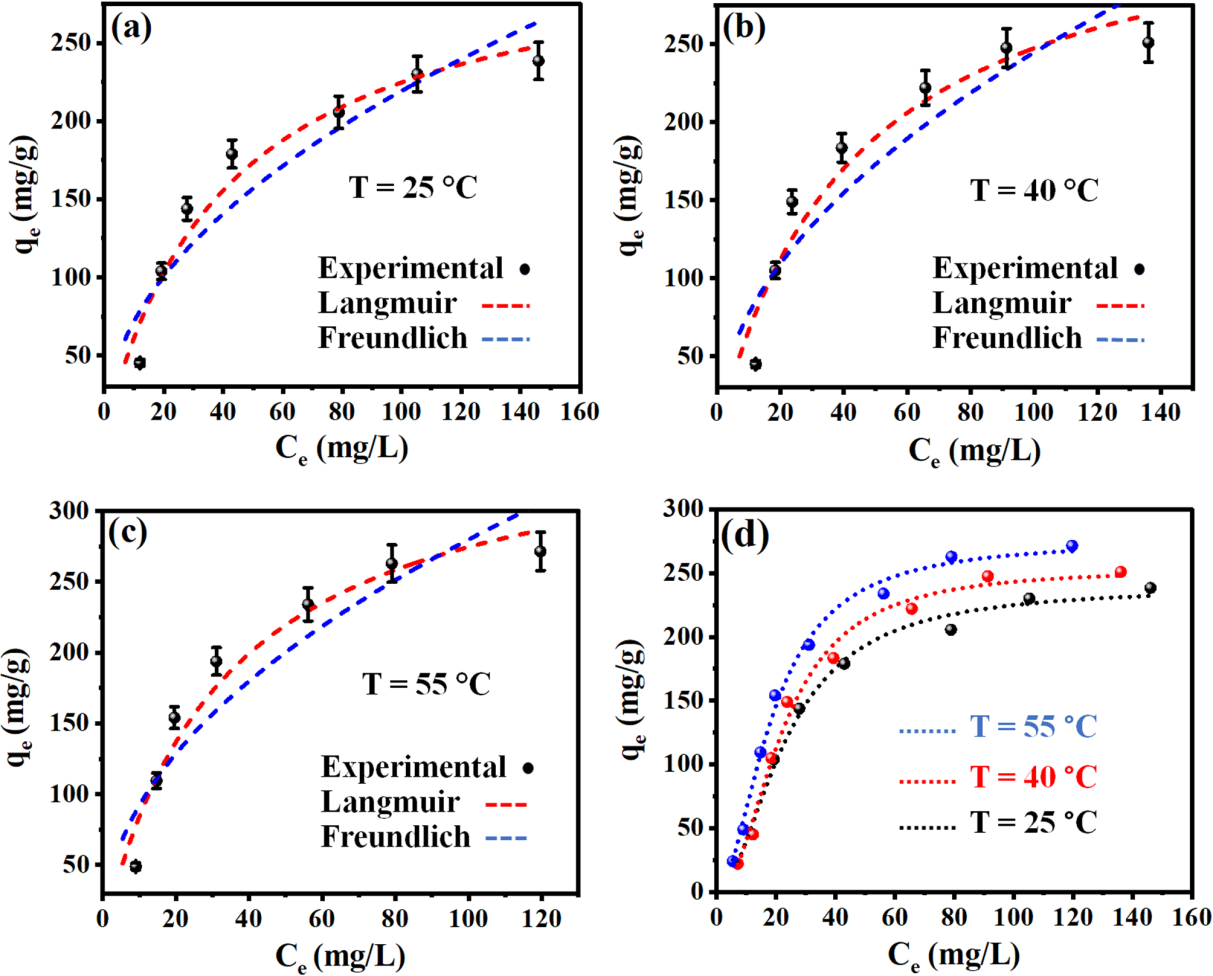
Nonlinear regression graphs of the isotherm (**a**–**c**) and advanced statistical (**d**) models for CR uptake at different temperatures.

The Langmuir and Freundlich isotherm models were employed to characterize the elimination performance of CR onto OP-GPHC across three temperature regimes (25, 40, and 55 °C), with the modeling results presented in Fig. 9a–c and Table 7. Statistical evaluation using *R²* metrics conclusively established the Langmuir isotherm as the most accurate descriptor of CR adsorption performance across most investigated temperature regimes, outperforming Freundlich model. As a result, CR molecules were eliminated via the OP-GPHC adsorbent at uniformly distributed active adsorption sites. This interaction between the OP-GPHC surface and the targeted contaminant led to the establishment of a monolayer consisting of CR molecules. The Langmuir *q*_*max*_ values at temperatures of 25, 40, and 55 °C were determined to be 318.96, 353.96, and 367.00 mg/g, as detailed in Table 7. The observed temperature-dependent increase in *q*_*max*_ values provides compelling evidence for the endothermic nature of CR adsorption onto the OP-GPHC adsorbent. This implies that the CR/OP-GPHC interactions are significantly more effective at elevated temperatures, thereby optimizing the adsorption efficiency. The *K*_*F*_ of Freundlich model exhibited temperature-dependent enhancement, increasing from 23.57 to 30.24 (mg/g)(mg/L)^−1/n^, thermodynamically proving the endothermic nature of the adsorption process. Furthermore, the observed 1/nF values below unity (1/*n*_*F*_ < 1.0) demonstrated favorable OP-GPHC reactions even at trace adsorbate concentrations, indicating strong surface affinity^59^. Furthermore, the performance of the raw precursors (RT, RG, and OSW-AC) was markedly inferior to the fabricated adsorbent, exhibiting adsorption capacities of only 45.34, 58.05, and 137.77 mg/g at an initial concentration of 150 mg/L. This pronounced difference confirms the superior efficacy and critical importance of the synthesized material.

### Interpretation of the CR adsorption mechanism via a statistical monolayer model

The adsorption isotherm data for CR were most accurately described by the advanced monolayer (AML) model, as detailed in Table S1 of the Supplemental Materials. This model demonstrated superior goodness-of-fit, with a coefficient of R² exceeding 0.98 and a *RMSE* between 6.32 and 8.32. As illustrated in Fig. 9d, the AML model effectively captures the adsorption behavior of CR across all tested temperatures, demonstrating significant model fit consistency. To elucidate the adsorption mechanisms of CR, critical parameters were evaluated, including the number of adsorbed molecules or ions per active site (*n*), adsorption density (*N*_*M*_), saturation uptake capacity (Q_sat_), and adsorption energies (*ΔE*)^59^.

**Table 7:** Equilibrium adsorption parameters derived from classical isotherm and advanced statistical models for CR sequestration by OP-GPHC binder.

Traditional model	T (^o^C)		Parameters$$\:{K}_{F}$$1/*n*_F_$$\:\:\:\:\:\:\left(\right(\mathrm{m}\mathrm{g}/\mathrm{g})$$$$\:{(\mathrm{m}/\mathrm{L})}^{-1/n})\:\:\:\:\:\:\:\:\:\:\:\:\:\:\:\:\:\:\:\:\:\:\:\:\:\:\:\:\:\:\:\:\:\:\:\:$$		$$\:{R}^{2}$$
Freundlich	25		23.57	0.484	0. 891
	40		24.50	0.500	0. 869
	55		30.24	0.483	0.886
			$q_{{\max }} (mg/g)$	$K_{L} (L/mg)$	
Langmuir	25		318.96	0.024	0. 959
	40		353.96	0.232	0.945
	55		367.00	0.029	0.960

Advanced monolayer model					
*T* (ºC)$\:n$	$\:{N}_{M}\:$(mg/g)	*R* ^*2*^	$\:\varDelta\:E\:$(kJ/mol)	$\:{Q}_{sat}\:$(mg/g)	*RMSE*
25 1.88	127.45	*0.995*	19.25	239.61	*6.32*
40 2.07	122.67	*0.990*	20.34	235.93	*8.32*
55 1.86	147.93	*0.993*	21.46	275.15	*6.44*

#### Statistical steric factors (n, N_M_, and Q_sat_)

The interaction mechanism and adsorption configuration of CR dye on the OP-GPHC adsorbent can be elucidated through the *n* parameter. This parameter characterizes the adsorption process between CR molecules and the functional groups of the OP-GPHC binder, as determined through three distinct analytical measurements. The removal of CR onto the multiple functional groups of the OP-GPHC adsorbent exhibits a horizontal orientation and a multi-docking removal mechanism, as indicated by the first measurement (where the n parameter is less than 0.5). The second adsorption regime, characterized by an n parameter value between 0.5 and 1, demonstrates that CR molecules are adsorbed by the OP-GPHC in a mixed geometry, incorporating both vertical and horizontal orientations. Eventually, the third adsorption regime, where the n parameter exceeds 1, suggests a multimolecular adsorption mechanism with a predominantly vertical orientation on the OP-GPHC surface.

Figure S3a demonstrates how the *n* parameter changes as solution temperature rises during CR adsorption, while Table 7 provides the associated quantitative data. The statistically determined *n* parameter values for CR adsorption were 1.88, 2.07, and 1.86 at 25 °C, 40 °C, and 55 °C, respectively. The experimental findings indicate a consistent adsorption mechanism and molecular orientation across the studied temperature range (25–55 °C), as evidenced by n parameter values consistently exceeding unity. Further analysis revealed that CR/OP-GPHC interactions maintained a vertical adsorption geometry through a multimolecular interaction mechanism throughout the investigated thermal conditions. Furthermore, the self-association and agglomeration of CR molecules in the aqueous phase prior to adsorption were identified as a preliminary step that enhances the overall removal efficiency and adsorption capacity. This conclusion is supported by *n* parameter values between 1.86 and 2.07. Notably, thermal variations across the studied range (25–55 °C) demonstrated no significant influence on either the spatial configuration of adsorbed species or their binding interactions with the adsorption sites of OP-GPHC binder.

The active site density (*N*_*M*_) of OP-GPHC exhibits temperature-dependent behavior, as shown in Fig. S3a across the 25–55 °C range. Quantitative evaluation of the *N*_*M*_ values (Table 7) revealed distinct adsorption capacities of 127.45, 122.67, and 147.93 mg/g for CR adsorption across the tested temperature gradient. The observed increase in *N*_*M*_ values for CR adsorption at elevated solution temperatures suggests potential activation of additional binding sites on the OP-GPHC adsorbent, which likely enhanced CR removal efficiency^25^. Furthermore, the results demonstrate that increasing the n parameter consistently reduces *N*_*M*_ at each tested temperature^65^. This temperature-dependent behavior of the *N*_*M*_ parameter correlates well with the endothermic characteristics of the CR elimination process.

In the context of the accepted AML model, the saturation adsorption capacity, denoted as the *Q*_*sat*_ parameter, is determined using the equation: *Q*_*sat*_ = *n* * *N*_*M*_^59^. The accurate assessment of this parameter is essential for acquiring valuable insights into the effectiveness of the evaluated adsorbent. Notably, a rise in the temperature of the aqueous solution from 25 to 55 °C led to an enhancement of the CR saturation uptake capacities, rising from 239.61 mg/g to 275.15 mg/g, as demonstrated in Fig. S3b and confirmed in Table 7. The enhanced removal capacities observed at higher temperatures likely stem from robust intermolecular interactions between CR dye molecules and specific functional groups on the OP-GPHC adsorbent surface. This thermal dependence further corroborates the endothermic nature of the adsorption process involving CR molecules and the active sites of OP-GPHC.

#### Adsorption energy

The adsorption behavior of CR on OP-GPHC can be effectively characterized through determination of the adsorption energy (*ΔE*). Theoretical analysis based on the AML model framework enabled quantification of the adsorption energies required for monolayer formation of CR across multiple temperature conditions^66^.

5 $$\:{\mathrm{C}}_{1/2}={\mathrm{C}}_{\mathrm{s}}{\mathrm{e}}^{-\:\frac{\varDelta\:\mathrm{E}}{\mathrm{R}\mathrm{T}}}$$

Here, $\:{C}_{s}$ represents the solubility limit of either CR, while $\:{C}_{1/2}$ denotes the half-saturation concentration.

Figure S3b, along with Table 7, illustrate the variation of ΔE with temperature (25–55 °C). The positive *ΔE* values confirm the endothermic nature of CR/OP-GPHC interactions^66^. Moreover, the *ΔE* for CR consistently exceeded 20 kJ/mol across most experimental temperatures, indicating that chemical interactions predominantly govern the adsorption mechanism. Thermodynamic analysis revealed a concurrent increase in both *ΔE* and Q_sat_ across the 25–55 °C temperature range, demonstrating synchronized energetic and steric parameter behavior. This correlation confirms that the adsorption energy plays a pivotal part in augmenting the removal capacities of CR by the OP-GPHC adsorbent. Figure 14 illustrates the proposed interaction mechanism between CR molecules and the functional groups present in the synthesized OP-GPHC binder.

### Probable mechanistic interpretation of CR sequestration

**Fig. 10:**
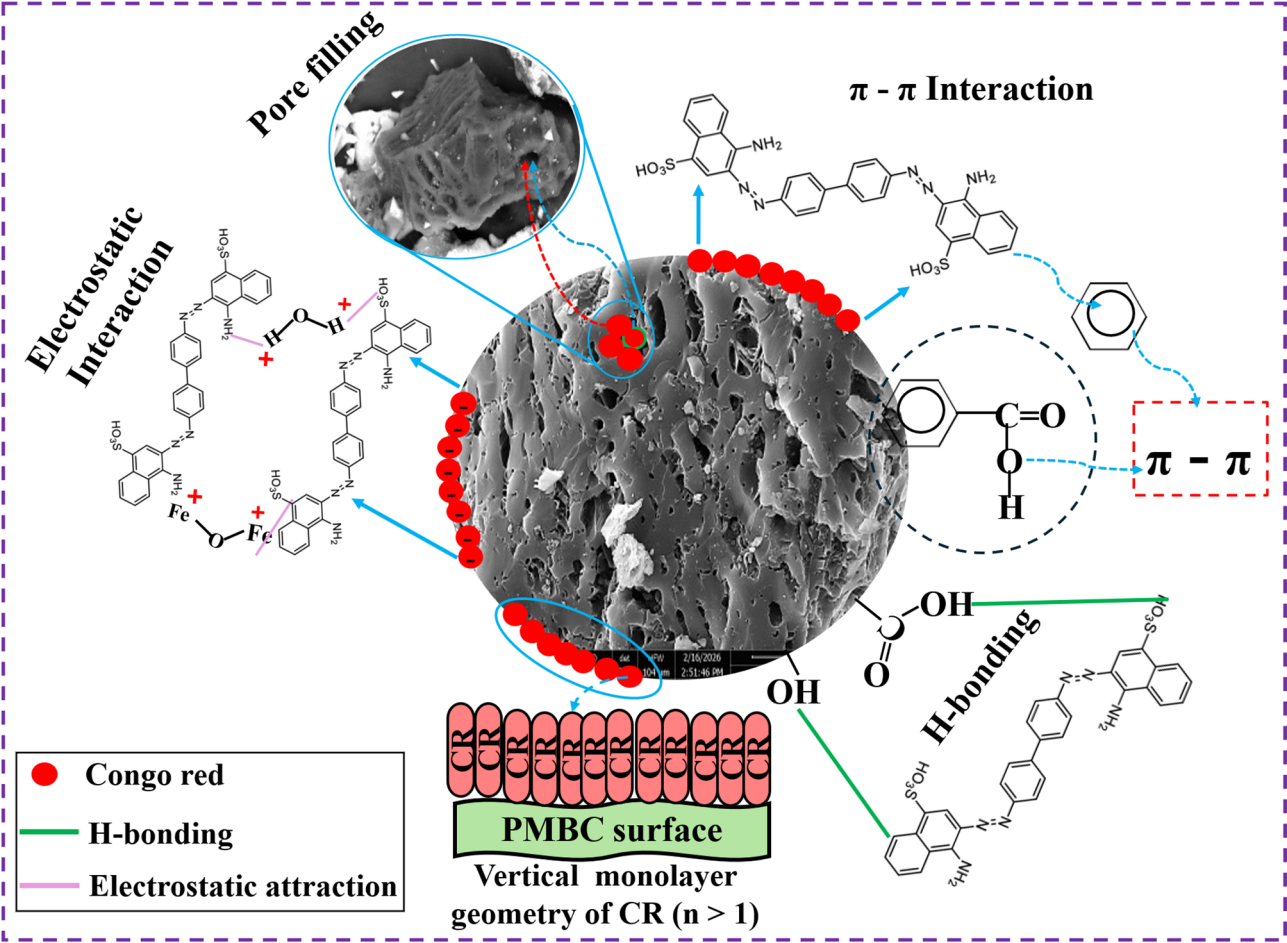
The possible removal mechanism of CR onto the OP-GPHC binder.

Figure 10 illustrates the multifaceted adsorption mechanisms governing CR sequestration by the OP-GPHC composite, which are quantitatively supported by multiple lines of experimental evidence. Spectroscopic evidence from FTIR analysis reveals characteristic shifts after CR loading (Si–O–Si: 1115.62 → 1098.43 cm⁻¹; O–H: 3430.74 → 3418.26 cm⁻¹) and new peaks at 1578.32 cm⁻¹ and 1502.45 cm⁻¹, confirming hydrogen bonding between surface siloxane/hydroxyl groups and the amino/azo functionalities of CR. Energetic evidence from statistical physics modeling (ΔE = 19.25–21.46 kJ/mol, Table 7) falls within the range characteristic of physisorption (hydrogen bonding, π–π stacking, and van der Waals forces), while approaching the lower threshold of chemisorption, consistent with the involvement of electrostatic interactions. pH-dependent evidence shows maximum removal (99.2%) at pH 2.0, well below the pHpzc (≈ 7), where protonated Al–OH₂⁺ and Si–OH₂⁺ sites electrostatically attract the anionic sulfonate groups (–SO₃⁻) of CR, while the sustained removal (> 80%) at pH 8–9 confirms the contribution of pH-independent π–π stacking between CR’s aromatic rings and the graphitic domains of OSW-AC, supported by BET analysis (Type IV isotherm, 38.07 m²/g, 20.14 nm pore diameter). Kinetic evidence from the concurrent fit of Elovich (R² = 0.995) and pseudo-first-order (R² = 0.998) models further corroborates a composite mechanism of chemisorption (complexation) and physisorption (π–π stacking, pore diffusion, hydrogen bonding, and electrostatic attraction) contributes significantly as the system approaches equilibrium. Collectively, this convergence of spectroscopic, energetic, pH-dependent, structural, and kinetic evidence establishes that CR adsorption onto OP-GPHC proceeds through a synergistic combination of electrostatic attraction, hydrogen bonding, π–π stacking, quantitatively linking the illustrative mechanism in Fig. 10 to the experimental data throughout this study.

### Thermodynamic studies for CR adsorption

#### Entropy

**Fig. 11:**
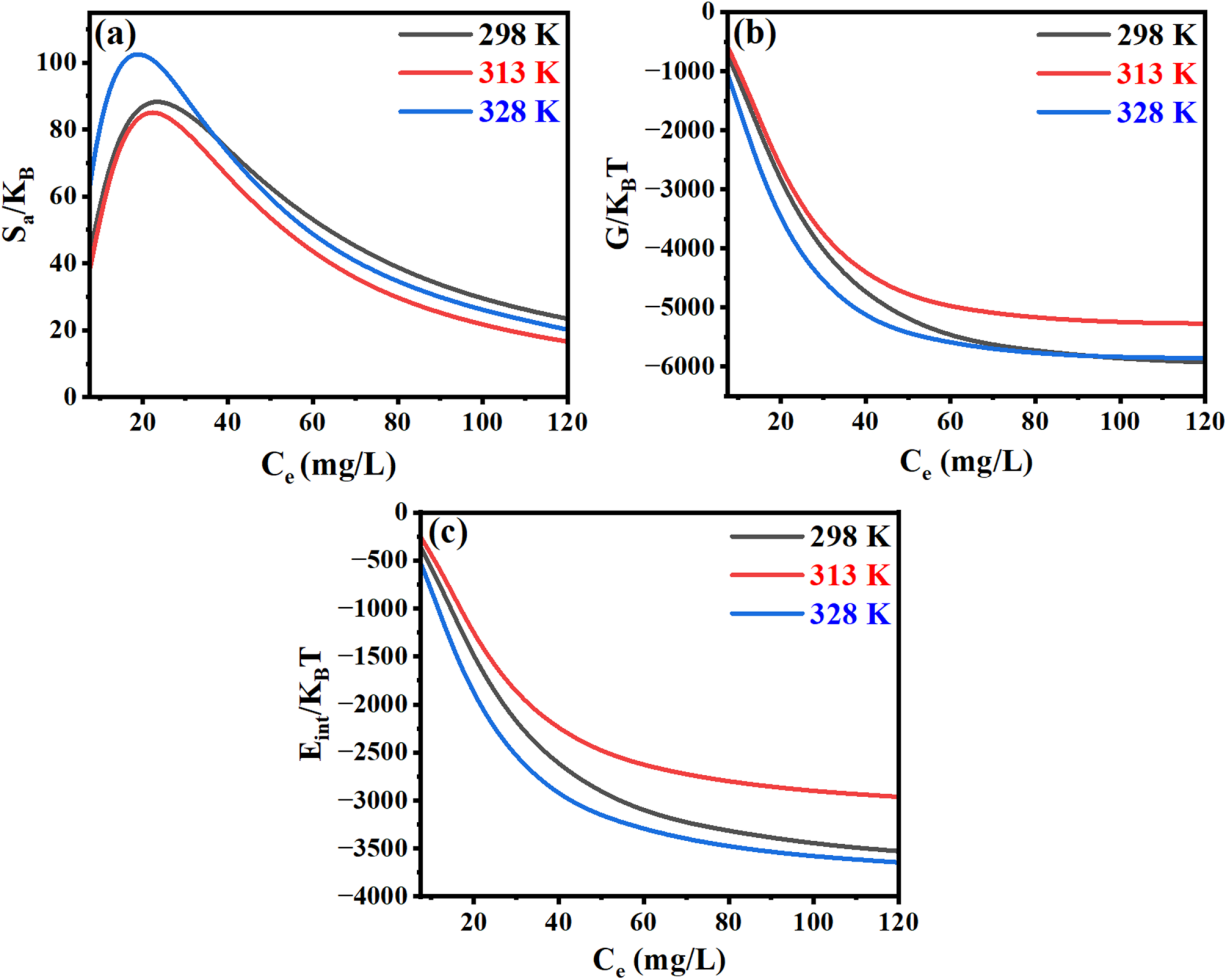
Thermodynamic assessment of CR adsorption on OP-GPHC: (**a**) entropy, (**b**) gibbs free energy, and (**c**) internal energy.

Figure 11a illustrates the dependence of entropy on CR concentration. A pronounced increase in thermodynamic entropy - quantitatively characterizing system disorder - is observed with elevating CR concentration, achieving maximal values prior to reaching half-saturation conditions. The OP-GPHC binder surface exhibits numerous high-energy adsorption sites capable of effectively sequestering CR molecules from aqueous solutions. This interfacial activity drives the initial rise in system entropy, reflecting increased molecular disorder upon adsorption. Conversely, a marked reduction in entropy beyond a critical CR concentration threshold signifies a transition toward a more ordered molecular configuration. This increased order results from the progressive saturation of available adsorption sites on the OP-GPHC surface^38,67^.

#### Gibbs free energy

The variation of Gibbs free energy as a function of CR concentration is illustrated in Fig. 11b. Notably, the Gibbs free energy attains its peak magnitude during the initial phase of adsorption, demonstrating a pronounced affinity between the OP-GPHC adsorbent and CR molecules^3,38^. Furthermore, increasing adsorbate concentration resulted in a progressive reduction in the Gibbs free energy magnitude at the CR/OP-GPHC interface. The consistently negative values of Gibbs free energy confirmed the thermodynamically spontaneous nature of the CR adsorption process^38,68^.

#### Internal energy

Figure 11c presents the concentration-dependent evolution of internal energy in the adsorption system. The consistently negative internal energy values provide thermodynamic confirmation of spontaneous CR adsorption on the OP-GPHC substrate^69^.

### Regeneration of OP-GPHC binder

The reusability assessment of the OP-GPHC adsorbent, using 0.1 M HCl for regeneration (Fig. S4), indicates a high degree of stability across multiple cycles. The material retained a removal efficiency of 87.79% over five consecutive adsorption-desorption sequences, underscoring its robust and competitive potential for repeated application. An extreme proton competition, which the very high concentration of H⁺ ions in the desorption bath (0.1 M HCl, pH ~ 1) creates an intensely competitive environment. These excess protons can effectively screen or neutralize the negative charges on the already-adsorbed CR molecules, significantly weakening their anionic character and thus the electrostatic binding force. In addition, the surface charge alteration (potential), which in some adsorbent systems, an extremely low pH can also begin to protonate specific functional groups on the adsorbent itself, potentially reducing its net positive charge or even driving it toward a point of zero charge, further destabilizing the electrostatic bond. The OP-GPHC binder demonstrates exceptional reusability for CR removal, maintaining stable adsorption capacity across multiple cycles, while its composition and sustained performance validate its potential for industrial-scale implementation in wastewater treatment, offering an economically viable and sustainable solution for organic pollutant remediation.

### Post-use valorization strategies and circular economy perspectives

Following the assessment of reusability, a critical consideration for sustainable adsorbent design is the end-of-life management of the spent material. Unlike conventional biosorbents that often face disposal challenges, the OP-GPHC composite offers viable post-use valorization pathways that align with circular economy principles.

One promising route is the thermochemical conversion of the spent adsorbent via pyrolysis. Given that the OP-GPHC matrix contains a substantial fraction of olive seed waste-derived activated carbon embedded within a geopolymeric framework, the exhausted material—now laden with adsorbed CR dye—can be subjected to controlled pyrolysis. This process would serve a dual purpose: (i) the thermal degradation of the retained organic dye molecules, potentially recovering energy, and (ii) the transformation of the geopolymeric residue into a stable, carbon-enriched solid product. Such a derivative could find secondary applications as a low-cost catalyst support, a filler in construction materials, or as a precursor for regenerated adsorbents, thereby extending the material’s lifecycle and minimizing waste disposal burdens. This approach is particularly advantageous compared to many biosorbents, which, after use, typically require landfilling or incineration without added value recovery^9,70^.

Furthermore, beyond the regeneration of the adsorbent itself, the recovered CR dye from the desorption process represents an untapped resource. The anionic nature and aromatic structure of CR, which contribute to its environmental persistence, also render it potentially valuable in other industrial contexts. Notably, azo dyes such as CR have been demonstrated to function as effective corrosion inhibitors for metals in aggressive environments. The presence of heteroatoms (nitrogen, sulfur, oxygen) and conjugated π-electron systems in CR molecules enables strong adsorption onto metal surfaces, forming protective films that mitigate corrosion. This strategy not only eliminates a secondary waste stream but also creates economic value from what would otherwise be a pollutant, embodying the principles of waste-to-resource conversion^3,71^.

### Comparative performance evaluation of competing adsorbents

Table S2 presents the comparative adsorption performance of OP-GPHC against conventional adsorbents, demonstrating a superior maximum CR adsorption capacity of 367.00 mg/g. These findings establish OP-GPHC as a cost-effective, high-performance material for azo dye remediation, with direct applicability in industrial wastewater treatment systems for CR-contaminated effluents.

### Cost estimation of OP-GPHC adsorbent

The commercial feasibility of adsorbent materials for wastewater remediation hinges significantly on their production economics, encompassing both energy expenditure and chemical reagent costs during synthesis. Table 8 summarizes the production economics OP-GPHC, revealing a manufacturing cost of 0.032 USD per gram while demonstrating superior cost-efficiency relative to conventional adsorbents. Furthermore, the economic analysis compiled in Table S3 shows that OP-GPHC demonstrates significantly greater cost-effectiveness when compared to conventional water treatment adsorbents reported in the literature.

**Table 8:** Cost analysis of the fabricated OP-GPHC adsorbent.

Material	Purchased quantity	Total purchase cost (USD)	Purchasing cost (USD/g or mL)	Used quantity (g or mL)	Cost of used quantity (USD)
OSW	300 g	1.2	0.0012	200	0.24
RT	2 kg	0.394	0.000197	500	0.0985
RG	2 kg	0.591	0.0002955	500	0.1477
NaOH	500 g	15.76	0.0315	75	2.3625
H_2_O_2_	1L	9.85	0.00985	100	0.985
HCl	500 mL	17.73	0.03546	70	2.4822

Equipment	Time (H)	Power consumption (kWh/Kg)	Unit cost of power (USD/kWh)	Cost
Calcination	3	5	0.12	1.8
Drying	48	2	0.12	11.52
Mixer	0.1	1	0.12	0.012
		Total yield cost = 19.65 USD For 613.25 g	Total yield cost 0.032 USD/g	

### Scale-up cost estimation

To forecast the potential for scaling the OP-GPHC composite for CR uptake, the batch adsorption results were modeled mathematically. The Langmuir isotherm, which provided the most accurate representation of the equilibrium conditions, was employed to characterize the adsorption mechanism. The parameters derived from this isotherm were subsequently incorporated into a mass transfer balance, yielding the predictive scaling relationship detailed in Eq. (6)^11,72^.

6 $$\:\frac{m}{V}=\frac{({C}_{0}-{C}_{e})(1+{K}_{L}{C}_{e})}{{C}_{e}{q}_{max}\:{K}_{L}}$$

This predictive scaling relationship is fundamentally governed by the proportional link between the quantity of OP-GPHC adsorbent (*m*, g) and the volume of CR solution (*V*, L) it can purify. The algorithm calculates the contaminant removal by analyzing the initial and final solute concentrations (C_e_ and C_e_, mg/L), with the Langmuir parameters for binding affinity (*K*_*L*_) and maximum adsorption capacity (*q*_*max*_) serving as the core thermodynamic variables. This equation assumes plug flow behavior based on batch data, not a validated continuous system design. This approach aligns with established chemical engineering principles, whereby the design of full-scale adsorption units often proceeds from laboratory-derived batch studies. This pragmatic convention is well-documented, even though data from continuous-flow configurations more accurately reflect real-world process dynamics^73^.

**Fig. 12:**
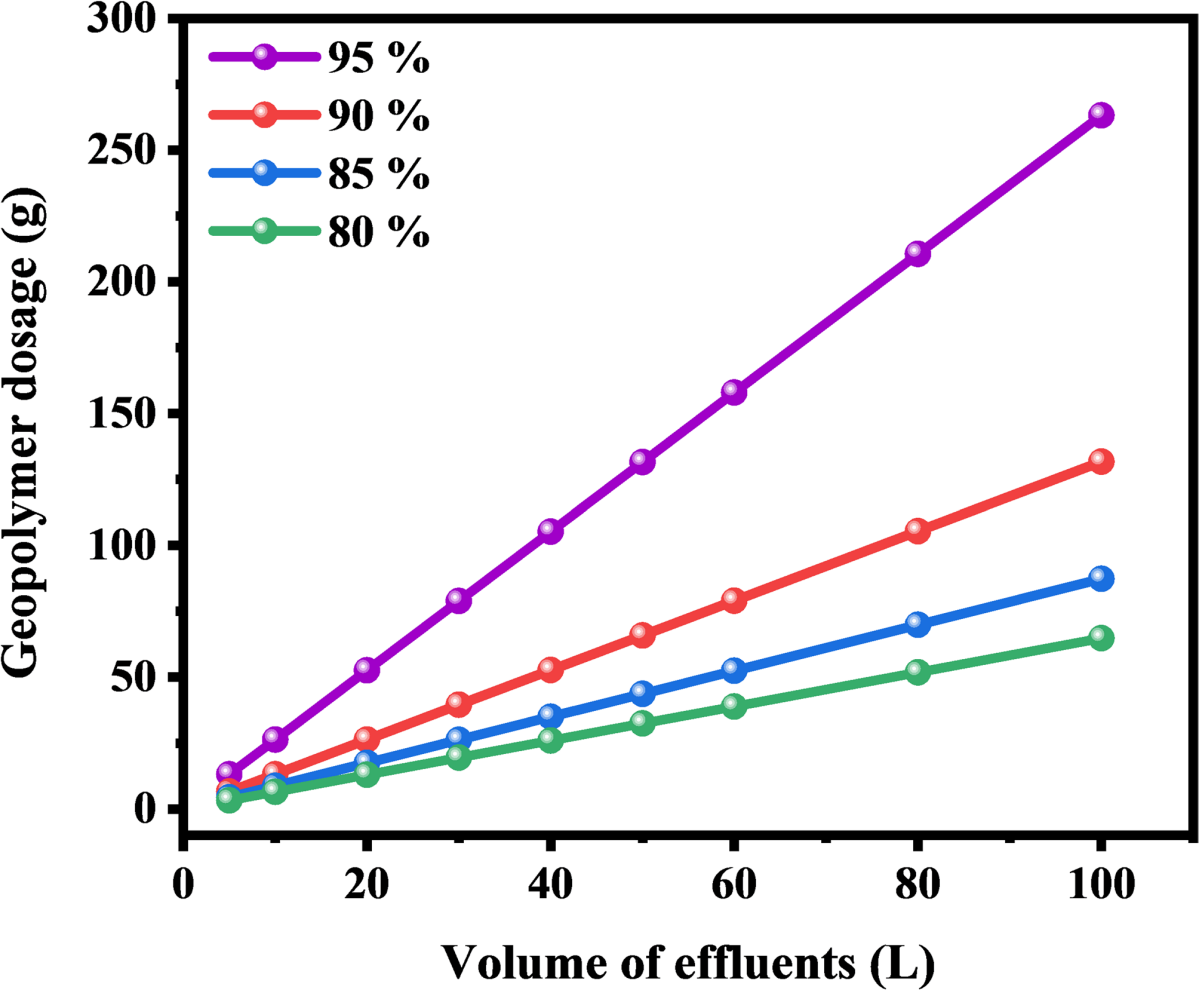
The amounts of OP-GPHC adsorbent required for different CR removal efficiencies (80–95%) from polluted solutions.

A quantitative assessment of the scale-up potential was conducted by projecting the requisite adsorbent mass for treating effluent volumes from 5.0 to 100.0 L, targeting removal efficiencies of 85% to 95%. These projections, calculated using Eq. (6), assumed constant operational parameters: an initial pollutant concentration (*C₀*) of 50 mg/L, a temperature of 25 °C, a pH of 2, and a contact duration of 60 min. Figure 12 consolidates the results of this scaling analysis. The composite’s capacity for repeated use distributes its cost over multiple applications, yielding an amortized expense of nearly $0.032/g. In a hypothetical 100-liter treatment scenario designed for 95% contaminant sequestration, the process would initially demand 263.24 g of virgin adsorbent. By adopting a regeneration strategy, the financial burden of treating a subsequent 100-liter volume drops to approximately $1.68, while still maintaining a removal efficiency of 87.79%. A scaled operation to treat 100 L of effluent for 95% sequestration requires an initial 263.24 g of unused adsorbent. However, employing a regeneration protocol for subsequent treatment cycles reduces the cost for an equivalent volume to about $1.69, while still securing a removal efficiency of 86.68%. These financial estimates are founded on dosage requirements determined in prior work, where key operational parameters such as pH, initial concentration, and reaction time were held constant at their optimized levels^72^. Furthermore, the scaling relationship defined in Eq. (6) indicates that the mass of OP-GPHC needed to treat 100 L with a *C₀* of 50 mg/L is a direct function of the target decontamination efficiency. The computed data, displayed in Fig. 12, quantify this relationship; achieving 80, 85, 90, and 95% eimination of CR necessitates adsorbent masses of 64.83, 87.40, 131.75, and 263.24 g, respectively. The compelling balance achieved between treatment expense and operational performance confirms the adsorbent’s strong suitability for practical implementation in water remediation.

## Challenges, assumptions, and future prospects

Despite the promising performance of the OP-GPHC composite, its translation from laboratory-scale discovery to practical deployment requires acknowledging several inherent assumptions and addressing key limitations.

### Assumptions

Idealized experimental matrix: The adsorption experiments were conducted using tap water spiked with CR to establish fundamental mechanistic understanding. This approach assumes that the behavior observed in a simplified matrix is representative of performance in real, complex industrial wastewaters containing competing organic matter, suspended solids, and fluctuating ionic backgrounds.

Scale-up linearity: The scaling calculations (Sect. 3.13) assume that equilibrium and kinetic parameters derived from batch studies are directly transferable to continuous or large-scale batch systems without significant deviation due to changes in hydrodynamics, mixing efficiency, or mass transfer regimes.

Regeneration stability: The economic assessment assumes that the adsorbent’s performance over five cycles (87.8% retention) will be sustained over many more cycles without accelerated fouling or structural degradation, which is typical for lab-scale assessments but may not fully capture long-term operational attrition.

### Limitations

Feedstock variability: The raw materials—glauconite, talc, and olive seed waste—were sourced from single geographic locations in Egypt. Variability in mineralogical composition (e.g., Fe₂O₃ content in glauconite, MgO content in talc) and organic matter content in the olive seed waste could influence the reproducibility and consistency of the final composite.

Narrow operational pH window: Maximum adsorption efficiency (99.2%) was achieved at pH 2.0, well below the pHpzc (≈ 7). Although the material retained > 80% removal at pH 8–9, this pronounced dependence on strongly acidic conditions may necessitate pre-acidification of real wastewaters (typically pH 6–9), adding operational complexity and cost.

Specificity of the adsorbate: While CR was selected as a model anionic azo dye, the adsorption performance and mechanistic conclusions may not be directly generalizable to other classes of pollutants, including cationic dyes, heavy metals, or emerging organic micropollutants, without further validation.

### Future prospects

Real-wastewater validation: Future studies must extend adsorption experiments to real industrial effluents containing competing ions, dissolved organic matter, and suspended solids to evaluate the material’s true operational performance, selectivity, and long-term stability. Pilot-scale continuous-flow studies are essential to validate the batch-derived scaling projections.

Surface engineering for pH broadening: To overcome the narrow optimal pH window, surface functionalization strategies—such as grafting with quaternary ammonium groups or incorporation of amphoteric modifiers—should be explored to enhance electrostatic attraction toward anionic dyes under circumneutral conditions, reducing or eliminating the need for pre-acidification.

Pilot-scale techno-economic and life-cycle assessment: The current cost analysis (0.032 USD/g) is based on laboratory-scale procurement. A comprehensive techno-economic assessment at pilot scale, coupled with life-cycle analysis (LCA) to quantify environmental impacts across production, use, and end-of-life phases, will be critical for establishing the material’s competitive positioning against existing commercial adsorbents.

## Conclusions

An innovative geopolymeric hybrid composite of alkali-activated glauconite/talc (OP-GPC) was synthesized as a sustainable platform for water remediation. Following detailed characterization, its potential was confirmed through the effective adsorption of anionic CR dye. Process optimization via Box-Behnken design established ideal parameters (i.e., pH 2, 0.07 g adsorbent dosage, and 77.5 min contact time), yielding a peak removal efficiency of 99.16% for a CR concentration of 150 mg/L. Subsequent analysis of the sequestration process showed strong conformity with the Langmuir isotherm and PFO kinetic models. Deep insights from statistical physics modeling indicated that multiple CR molecules associate with each active site on the adsorbent surface. This phenomenon is indicative of a multi-layer adsorption mechanism, characterized by a preferential vertical orientation of the dye molecules, which remained consistent across the investigated temperature spectrum. Thermal activation was found to substantially augment the adsorption capacity of the OP-GPHC adsorbent, resulting in an increase from 318.96 mg/g at 298 K to 367.00 mg/g at 328 K. Thermodynamic evaluations confirmed the endothermic and spontaneous nature of the process, with calculated adsorption energies ranging from 19.25 to 21.46 kJ/mol. Consequently, this research establishes the composite as a cost-effective and ecologically sound solution for the efficient removal of carcinogenic CR from aqueous environments.

## Supplementary Information

Below is the link to the electronic supplementary material.

Supplementary Material 1

## Data Availability

The datasets used and/or analysed during the current study available from the corresponding author on reasonable request.
