# Supplementary material for: Sustainable one-part geopolymeric hybrid composite derived from glauconite, talc, and olive seed waste–based activated carbon for Congo red adsorption
Source: Sci Rep. 2026 May 22;16:15991. doi: 10.1038/s41598-026-50411-7 (PMC13197495; doi:10.1038/s41598-026-50411-7)
Supplement: Supplementary file 1 — Supplementary Material 1 [file 41598_2026_50411_MOESM1_ESM.docx]

**Sustainable one-part geopolymeric hybrid composite derived from glauconite, talc, and olive seed waste–based activated carbon for Congo red adsorption**

**Supplementary Material**

*S1. Kinetic modeling of CR adsorption onto OP-GPHC*

To interpret the kinetic behavior of the adsorption process, four established models were applied: the pseudo-first-order (PFO), pseudo-second-order (PSO), intra-particle diffusion (IPD), and Elovich equations [1-4]. The specific mathematical expressions for these models are provided in the supporting Eqs. (S1) through (S4), where *qₜ* (mg/g) and *qₑ* (mg/g) represent the adsorption capacity at time *t* (min) and at equilibrium, respectively.

$q_{t}=q_{e} e^{-k_{1}t}$ S1

where *k₁* (min⁻¹) is the pseudo-first-order rate constant.

$q_{t} =\frac{q_{e}^{2} k_{2} t}{q_{e}k_{2} t+1}$ S2

where *k₂* (g·mg⁻¹·min⁻¹) is the pseudo-second-order rate constant.

$q_{t}=k_{p} t^{1/2}+C$ S3

where *kₚ* (mg·g⁻¹·min⁻⁰·⁵) is the intra-particle diffusion rate constant, and *C* (mg·g⁻¹) is a constant related to the boundary layer thickness.

*q_t_ =* $\frac{1}{\beta} Ln(\alpha\beta t+1)$ S4

where *α* (mg·g⁻¹·min⁻¹) is the initial adsorption rate, and *β* (g·mg⁻¹) is the desorption constant related to the extent of surface coverage.

*S2. Conventional isotherm models of CR adsorption onto OP-GPHC*

The mathematical formulations of both Langmuir [5] and Freundlich [6] isotherms were presented through non-linear equations, as detailed in Eqs. S5 and S6 respectively.

$q_{e}=\frac{q_{\text{max }K_{L}C_{e}}}{\left( 1+K_{L}C_{e} \right)}$ S5

where *qₑ* (mg/g) is the equilibrium adsorption capacity, *Cₑ* (mg/L) is the equilibrium concentration of CR in solution, *qₘₐₓ* (mg/g) is the maximum adsorption capacity, and *Kₗ* (L/mg) is the Langmuir constant related to the affinity of binding sites.

$q_{e}=K_{F}$ ${C_{e}}^{1/n}$ S6

where *K_F_* (mg/g (mg/L)⁻¹/ⁿ) is the Freundlich constant indicating adsorption capacity, and *n* (dimensionless) is the Freundlich exponent indicating adsorption intensity.

The relative adequacy of each model was ascertained by applying Eqs. (S7)-(S9) and comparing the resulting coefficient of determination (*R²*), Chi-square statistic (*χ²*), and root mean square error (*RMSE*) [7-9].

$R^{2}=1-\frac{\sum\left( q_{e,\exp}-q_{e,\mathrm{cal}} \right)^{2}}{\sum\left( q_{e,\exp}-q_{e,\mathrm{mean}} \right)^{2}}$ S7

$\chi^{2}=\sum\frac{\left( q_{e,\exp} - q_{e,\mathrm{cal}} \right)^{2}}{q_{e,\mathrm{cal}}}$ S8

$RMSE=\sqrt{\frac{\sum_{i=1}^{m} \left( Q_{i \mathrm{cal}}-Q_{i \exp} \right)^{2}}{p-1}}$ S9

where *qₑ,ₑₓₚ* (mg/g) and *qₑ,𝒸ₐₗ* (mg/g) are the experimental and calculated adsorption capacities, respectively; *qₑ,ₘₑₐₙ* (mg/g) is the mean of the experimental capacities; *Qᵢ,𝒸ₐₗ* and *Qᵢ,ₑₓₚ* are the calculated and experimental values for the *i*-th observation; *m* is the number of observations; and *p* is the number of parameters in the model.

*S3. Advanced models of CR adsorption*

*S3.1.* *Advanced monolayer (AML)* *model*

The mathematical expression representing this monolayer adsorption mechanism is given by Eq. (S10) [7, 10].

$q_{e}=\frac{nN_{M}}{1+\left( \frac{c_{1/2}}{C_{e}} \right)^{n}}$ S10

where *qₑ* (mg/g) is the equilibrium adsorption capacity, *n* (dimensionless) is the number of CR molecules per active site, *Nₘ* (mg/g) is the density of active sites, *Cₑ* (mg/L) is the equilibrium concentration of CR, and *c₁/₂* (mg/L) is the half-saturation concentration (concentration at which half the active sites are occupied).

*S3.2.* *Advanced double-layer (ADL)* *model*

The computational procedure for deriving these energy values is fully detailed in Eq. (S11) [11, 12].

$q_{e}=nN_{M}\frac{\left( \frac{C_{e}}{c_{1}} \right)^{n}+2\left( \frac{C_{e}}{c_{2}} \right)^{2n}}{1+\left( \frac{C_{e}}{c_{1}} \right)^{n}+\left( \frac{C_{e}}{c_{2}} \right)^{2n}}$ S11

where *qₑ* (mg/g) is the equilibrium adsorption capacity, *n* (dimensionless) is the number of CR molecules per active site, *Nₘ* (mg/g) is the density of active sites, *Cₑ* (mg/L) is the equilibrium concentration of CR, and *c₁* (mg/L) and *c₂* (mg/L) are the characteristic concentrations related to the first and second adsorbed layers, respectively.

*S4.* *Thermodynamic studies*

*S4.1. Entropy*

This entropic value was computationally derived using the grand canonical partition function, *Z_gc*, integrated with the grand potential, *J*, as outlined in the subsequent derivation [13].

$J=-k_{B}T \ln Z_{\mathrm{gc}}=-\frac{\partial\ln Z_{\mathrm{gc}}}{\partial\beta}-T S_{a}$ S12

$\frac{S_{a}}{k_{B}}=-\beta\frac{\partial\ln Z_{\mathrm{gc}}}{\partial\beta}+\ln Z_{\mathrm{gc}}$ S13

where $k_{B}$ (J/K) is the Boltzmann constant, *T* (K) is the absolute temperature, $Z_{\mathrm{gc}}$is the grand canonical partition function, *β = 1/$k_{B}$ T*, *Sₐ* (J/K) is the adsorption entropy, and *J* (J) is the grand potential.

Entropy, as a thermodynamic parameter, was calculated as informed below [7].

$\frac{S_{\text{a}}}{k_{B}}=-\frac{nN_{M} \left( \frac{Ce}{c_{\frac{1}{2}}} \right)^{n} \ln\left[ \frac{Ce}{c_{\frac{1}{2}}} \right]}{\left( 1+\left( \frac{Ce}{c_{\frac{1}{2}}} \right)^{n} \right)}+ N_{M} \ln\left[ 1+\left( \frac{Ce}{c_{\frac{1}{2}}} \right)^{n} \right]$ S14

where *n* (dimensionless) is the number of CR molecules per active site, *Nₘ* (mg/g) is the density of active sites, *Cₑ* (mg/L) is the equilibrium concentration of CR, and *c₁/₂* (mg/L) is the half-saturation concentration.

*S4.2.* *Gibbs free energy*

The Gibbs free energy, calculated according to the derivation in Equation (S15), offers fundamental insight into the process's energetic feasibility and inherent favorability [13].

$G=\mu Q=k_{B}T\frac{nN_{M}\ln\left[ \frac{Ce}{z_{\mathrm{tr}}} \right]}{\left( 1+\left( \frac{c_{\frac{1}{2}}}{Ce} \right)^{n} \right)}$ S 15

where *G* (J) is the Gibbs free energy, *μ* (J/molecule) is the chemical potential of the adsorbed CR molecules, *Q* is the adsorbed quantity, *k_B_* (J/K) is the Boltzmann constant, *T* (K) is the absolute temperature, *n* (dimensionless) is the number of CR molecules per active site, *Nₘ* (mg/g) is the density of active sites, *Cₑ* (mg/L) is the equilibrium concentration of CR, *c₁/₂* (mg/L) is the half-saturation concentration, and *z_tr_* is the translational partition function of the CR molecule.

*S4.3. Internal energy*

The internal energy can be studied using the following technique [13].

$E_{\mathrm{int}}=-\frac{\partial\ln Z_{\mathrm{gc}}}{\partial\beta}+\frac{\mu}{\beta}\left( \frac{\partial\ln Z_{\mathrm{gc}}}{\partial\mu} \right)$ S16

where $E_{\mathrm{int}}$ (J) is the internal energy, $Z_{\mathrm{gc}}$is the grand canonical partition function, *μ* (J/molecule) is the chemical potential, *β = 1/k_B_ T*, and *k_B_* (J/K) is the Boltzmann constant.

$\frac{E_{\mathrm{int}}}{k_{B}T}=-\frac{N_{M}\left( \frac{Ce}{c_{\frac{1}{2}}} \right)^{n}}{\left( 1+\left( \frac{Ce}{c_{\frac{1}{2}}} \right)^{n} \right)}\left( n \ln\left[ \frac{Ce}{c_{\frac{1}{2}}} \right]-\ln\left[ \frac{Ce}{z_{\mathrm{tr}}} \right] \right)$ S17

where $E_{\mathrm{int}}$ (J) is the internal energy, *k_B_* (J/K) is the Boltzmann constant, *T* (K) is the absolute temperature, *Nₘ* (mg/g) is the density of active sites, *n* (dimensionless) is the number of CR molecules per active site, *Cₑ* (mg/L) is the equilibrium concentration of CR, *c₁/₂* (mg/L) is the half-saturation concentration, and *z_tr_* is the translational partition function of the CR molecule.

Fig. S1. pHpzc of OP-GPHC binder.


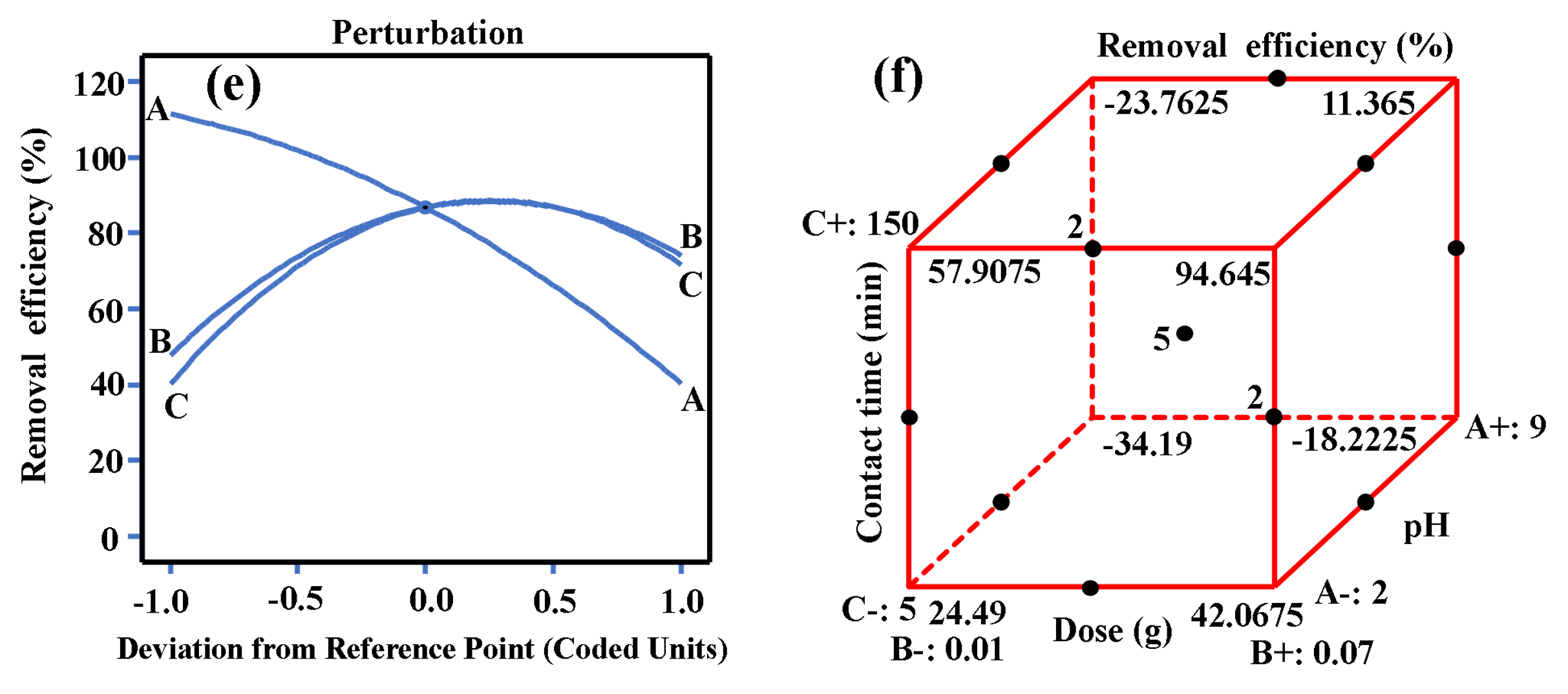


Fig. S2. (a) perturbation effects of key variables, and (b) cubic optimization profiles.

Table S1. *R*^2^ and *RMSE* values of the applied physics statical models.

| Advanced model | T (°C) |  |  |
| --- | --- | --- | --- |
| Monolayer | 25 | $R^{2}$ | 0.995 |
|  |  | *RMSE* | 6.32 |
|  | 40 | $R^{2}$ | 0.990 |
|  |  | *RMSE* | 8.32 |
|  | 55 | $R^{2}$ | 0.993 |
|  |  | *RMSE* | 6.44 |
| Double-layer | 25 | $R^{2}$ | 0.9492 |
|  |  | *RMSE* | 115.68 |
|  | 40 | $R^{2}$ | 0.9354 |
|  |  | *RMSE* | 100.51 |
|  | 55 | $R^{2}$ | 0.9236 |
|  |  | *RMSE* | 109.978 |


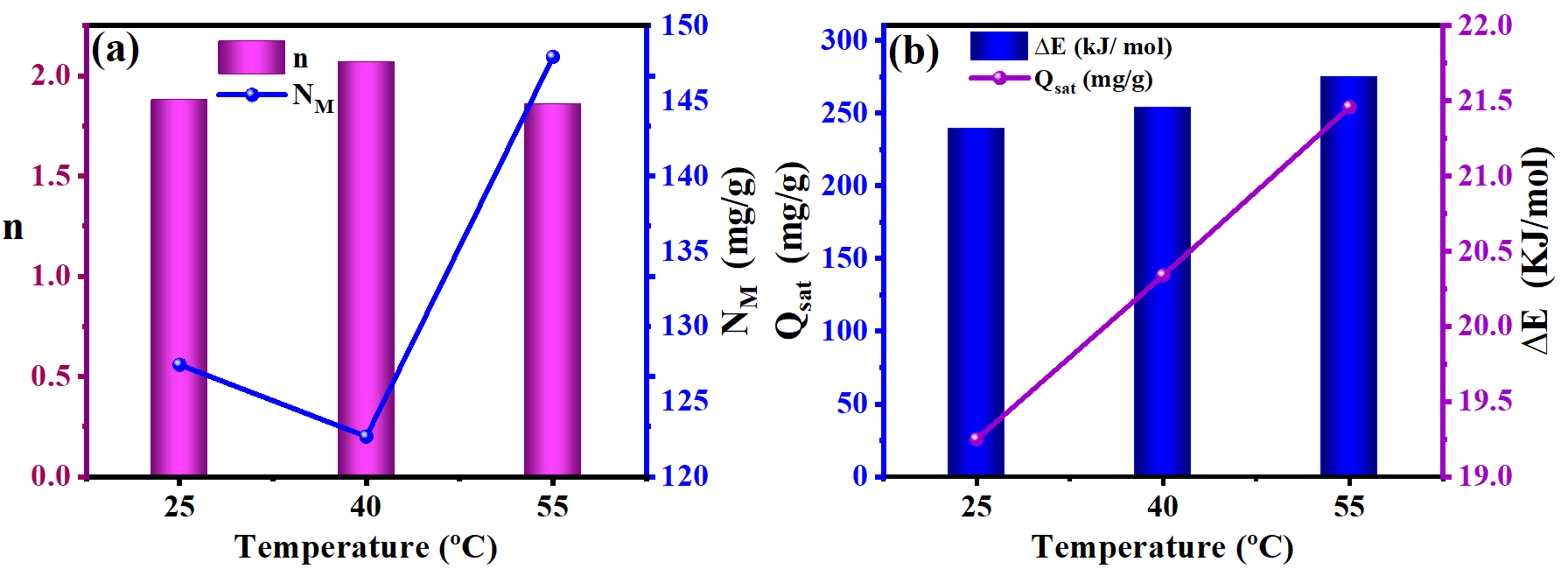


Fig. S3. The steric and energetic physicochemical parameters governing CR removal process across the investigated temperature rang.

Fig. S4. Recycling investigations of the OP-GPHC binder.

Table S2. *q_max_* of various adsorbents for CR uptake.

| **Adsorbents** | ***q_max_* _(_mg/g)** | **Reference** |
| --- | --- | --- |
| Red ilmenite ore | 78.53 | [14] |
| CS/PEG/ZnO | 256.41 | [15] |
| Acacia auriculiformis biochar | 130 | [16] |
| ZrO_2_/MgAl-LDH composites | 169.42 | [17] |
| BHP-MOFs | 176.06 | [18] |
| Cu-MOF | 119.76 | [19] |
| AFRL@AMNP | 218.2 | [20] |
| MgO decorated Fe_2_O_3_ nanorod | 200 | [21] |
| **OP-GPHC** | **367.00** | **Current study** |

Table S3. Cost comparison between the synthesized OP-GPHC and conventional adsorbents reported in existing literature.

| **Material used** | **Cost (USD/g)** | **Reference** |
| --- | --- | --- |
|  |  |  |
| PANI/LDH | 12.48 | [22] |
| Activated carbon | 1.33 | [23] |
| O-Pom/ LDH/PU | 0.927 | [24] |
| Graphene oxide/Titania | 0.1875 | [25] |
| OP-GPHC | 0.032 | **Current study** |

**References**

[1] L. S., ABOUT THE THEORY OF SO-CALLED ADSORPTION OF SOLUBLE SUBSTANCES, 24 (1898) 1–39.

[2] Y.S. Ho, G. McKay, Sorption of dye from aqueous solution by peat, Chemical Engineering Journal 70 (1998) 115–124.

[3] W.J. WeberJr., J.C. Morris, Kinetics of Adsorption on Carbon from Solution, Journal of the Sanitary Engineering Division 89 (1963) 31–59.

[4] M.H. Dehghani, A. Dehghan, A. Najafpoor, Removing Reactive Red 120 and 196 using chitosan/zeolite composite from aqueous solutions: Kinetics, isotherms, and process optimization, Journal of Industrial and Engineering Chemistry 51 (2017) 185–195.

[5] I. Langmuir, The constitution and fundamental properties of solids and liquids. Part I. Solids, J Am Chem Soc 38 (1916) 2221–2295.

[6] H.M.F. Freundlich, Over the adsorption in solution, J. Phys. Chem 57 (1906) 1100–1107.

[7] S.I. Yacoub, S.G. Saber, R.A.M. Ali, E.C. Lima, G.S. dos Reis, E. Al-Olayan, Y.F. Salama, M. Mobarak, M.K. Seliem, CTAB-modified alkali-activated binder derived from Favia corals and glass waste: A novel bio-based adsorbent for effective removal of Mn(VII) ions from aqueous solutions, Journal of Industrial and Engineering Chemistry 147 (2025) 406–421.

[8] A.S. Elshimy, M. Mobarak, J.S. Ajarem, S.N. Maodaa, A. Bonilla-Petriciolet, Z. Li, M.A. Korany, D.S. Ammar, D.G. Awad, S.A. Elberbash, M.K. Seliem, Sodium alginate-modified alkali-activated eggshell/Fe3O4 nanoparticles: A magnetic bio-based spherical adsorbent for cationic dyes adsorption, Int J Biol Macromol 256 (2024) 128528.

[9] M.K. Seliem, A.S. ElShimmy, M. Mobarak, A.Q. Seliem, A.M. Salah, Z.M. Almarhoon, Z. Li, Y.F. Salama, H.I. Bendary, A magnetic bio-based adsorbent prepared from Fe3O4 nanoparticles impregnated with diatom frustules and sodium alginate for methylene blue uptake: advanced modeling and mechanism, Sep Sci Technol 59 (2024) 99–111.

[10] M.A. Ali, A.M. Badawy, A.Q. Seliem, H.I. Bendary, E.C. Lima, M. Al-Dossari, N.S. Abd EL-Gawaad, G.S. dos Reis, M. Mobarak, A.M. Hassan, M.K. Seliem, Macroscopic and Microscopic Levels of Methylene Blue Adsorption on a Magnetic Bio-Based Adsorbent: In-Depth Study Using Experiments, Advanced Modeling, and Statistical Thermodynamic Analysis, Magnetochemistry 10 (2024) 91.

[11] M.A. Arif, H.A. Abdel-Gawwad, A.S. Elshimy, M.K. Seliem, M.A. Ali, S.N. Maodaa, K. Federowicz, M. Mobarak, H.I. Bendary, Y.F. Salama, M. Abd Elrahman, H. Soltan Hassan, Facile synthesis and characterization of metakaolin/carbonate waste-based geopolymer for Cr(VI) remediation: Experimental and theoretical studies, Inorganica Chim Acta 564 (2024) 121939.

[12] A.S.A.A. Abu Sharib, M. Mobarak, A.S. Elshimy, N. Al-arifi, Y.F. Salama, Z. Li, A.Q. Selim, M.K. Seliem, Facile and green fabrication of an effective and low-cost alkali-activated binder using carbonized limestone: Characterization, experiments, and statistical physics formalism for ibuprofen adsorption, Sustain Chem Pharm 41 (2024) 101701.

[13] A.S. Elshimy, H.A. Abdel Gawwad, A.A.A.A. Sharib, N.S.A. EL-Gawaad, Z.A.M. Al-Ahmed, A. Bonilla-Petriciolet, Z. Li, M. Mobarak, A.Q. Selim, M.K. Seliem, A new alkali-activated binder prepared from dolomite waste and diatom frustules: Insights into the mechanical performance and Mn(VII) treatment, J Environ Chem Eng 11 (2023) 110392.

[14] R. A. M. Ali, M. Mobarak, A. M. Badawy, E. C. Lima, M. K. Seliem, H. S. Ramadan, New insights into the surface oxidation role in enhancing Congo red dye uptake by Egyptian ilmenite ore: Experiments and physicochemical interpretations, Journal of Surfaces and Interfaces 26 (2021) 101316.‏

[15] S. Hussain, M. Salman, J. P. Youngblood, U. Farooq, S. Yasmeen, K. M. Al-Ahmary, M. Ahmed, Enhanced adsorption of Congo red dye by CS/PEG/ZnO composite hydrogel: Synthesis, characterization, and performance evaluation, Journal of Molecular Liquids 411 (2024) 125704.‏

[16] D. L. T. Nguyen, Q. A. Binh, X. C. Nguyen, T. T. H. Nguyen, Q. N. Vo, T. D. Nguyen, T. C. P. Tran, T. A. H. Nguyen, S. Y. Kim, T. P. Nguyen, J. Bae, I. T. Kim, Q. V. Le, Metal salt-modified biochars derived from agro-waste for effective congo red dye removal, Journal of Environmental Research 200 (2021) 111492.‏

[17] D. Brahma and H. Saikia, Synthesis of ZrO2/MgAl-LDH composites and evaluation of its isotherm, kinetics and thermodynamic properties in the adsorption of Congo red dye, Journal of Chemical Thermodynamics and Thermal Analysis 7 (2022) 100067.‏

[18] M. Wang, J. Zhou, M. Zhang, S. Li, L. Li, G. Chen, X. Huang, G. Liu, J. Wang, D. Xu, Facile fabrication of bioinspired hierarchical porous MOFs for selective adsorption of Congo red and Malachite green from vegetables and fruits juices, Journal of Environmental Technology & Innovation 30 (2023) 103132.‏

[19] Y. Wang, D. Ren, J. Ye, Q. Li, D. Yang, D. Wu, J. Zhao, Y. Zou, Highly efficient and targeted adsorption of Congo Red in a novel cationic copper-organic framework with three-dimensional cages, Journal of Separation and Purification Technology 329 (2024) 125149.‏

[20] H. Bai, Y. Feng, C. Zhu, P. Guo, J. Wang, Y. Zhou, L. Zhang, S. Li, J. Chen, Efficient adsorption of Congo Red (CR) dye onto novel lignin-based magnetic core-shell adsorbent: Synthesis, characterization and experimental studies, Journal of the Taiwan Institute of Chemical Engineers 164 (2024) 105689.‏

[21] S. K. Sahoo, J. P. Dhal, G. K. Panigrahi, Magnesium oxide nanoparticles decorated iron oxide nanorods: synthesis, characterization and remediation of Congo red dye from aqueous media, Journal of Composites Communications 22 (2020) 100496.‏

[22] M. Kamel, G. Abd El-fatah, A. Zaher, A. A. Farghali, S. I. Othman, A. A. Allam, R. Mahmoud, Cost-effective layered double hydroxides/conductive polymer nanocomposites for electrochemical detection of wastewater pollutants, Chinese Journal of Analytical Chemistry, 52(3) (2024) 100368.

[23] A. Syafiuddin, S. Salmiati, T. Hadibarata, M. R. Salim, A. B. H. Kueh, S. Suhartono, Removal of silver nanoparticles from water environment: experimental, mathematical formulation, and cost analysis, Journal of Water, Air, & Soil Pollution, 230(5), (2019) 102.

[24] R. Abdelazeem, W. Kamal, Z. E. Eldin, M. A. Roshdy, A. A. Allam, S. Saeed, R. Mahmoud, Exploring the potential of waste biomass of olive as an additive for layered double hydroxide/polyurethane as an effective and safe agent for the adsorption of drug residues: a bioremediation approach, Journal of Materials Advances, 5(22), (2024) 9092-9106.

[25] C. C. Fu, R. S. Juang, M. M. Huq, C. Te Hsieh, Enhanced adsorption and photodegradation of phenol in aqueous suspensions of titania/graphene oxide composite catalysts, Journal of the Taiwan Institute of Chemical Engineers 67 (2016) 338–345.
